# Triboelectric Sensory Intelligence: From Mechanical Information Encoding to In-Sensor Memory, Neuromorphic Computing, and Closed-Loop Interaction

**DOI:** 10.3390/s26216925

**Published:** 2026-11-01

**Authors:** Qiang He, Zhiping Feng, Liang He

**Affiliations:** 1 School of Mechanical Engineering, Sichuan University, Chengdu 610065, China; 2 School of Mechanical Engineering, Xihua University, Chengdu 610039, China; feng.zp@xhu.edu.cn; 3 School of Mechanical Engineering, State Key Laboratory of Intelligent Construction and Healthy Operation and Maintenance of Deep Underground Engineering, Sichuan University, Chengdu 610065, China; heliang007@tsinghua.org.cn

**Keywords:** triboelectric sensing, sensory intelligence, in-sensor computing, neuromorphic electronics, human–machine interaction

## Abstract

Triboelectric interfaces are progressing beyond self-powered sensing toward integrated sensory systems capable of encoding, retaining, transforming, and responding to mechanical information. Yet, the rapid convergence of triboelectric transduction with memory, neuromorphic devices, and intelligent interfaces has also blurred the distinctions between signal generation, state modulation, physical computation, and task-level intelligence. This Review establishes an information-centric framework for triboelectric sensory intelligence, in which mechanical interaction is traced through electrical encoding, signal–device coupling, internal state evolution, sensory processing, perception, and ultimately closed-loop action. Sensory representations based on amplitude, polarity, temporal structure, spatial patterns, material-dependent responses, and multimodal signals are considered in terms of encoding fidelity, separability, and robustness. Stateful mechanisms based on resistive switching, charge storage, ferroelectric polarization, and ionic or electrochemical dynamics are then related to sensory memory, temporal filtering, synaptic plasticity, event-based transformation, and other physical computational primitives. Emphasis is placed on distinguishing structural integration from processing location, device dynamics from implemented computation, and hardware processing from downstream software inference. The Review further links these principles to tactile and material perception, gesture and motion interfaces, multisensory integration, and sensory–motor feedback, while introducing system-level considerations of reproducibility, energy, latency, scalability, and generalization. Looking forward, reliable source–receiver co-design, adaptive processing, scalable heterogeneous integration, and closed-loop operation will be central to translating triboelectric interfaces from isolated device demonstrations into persistent and autonomous sensory systems.

## 1. Introduction

Mechanical interfaces generate rich information through contact, deformation and relative motion. Triboelectric nanogenerators (TENGs) convert these interactions into electrical signals that can be harvested as energy, interpreted as sensory waveforms or used to modulate the state of electronic devices [1]. These functions impose fundamentally different requirements: energy harvesting concerns useful electrical energy delivered to a load, sensing requires a reproducible relationship between mechanical input and electrical output, and sensory computing additionally requires a physical transformation that preserves, filters, stores or interprets task-relevant information. High electrical output alone therefore establishes neither accurate perception nor computation. Early flexible TENGs demonstrated mechanical-to-electrical energy conversion in deformable structures [2], while tribotronic devices subsequently exploited triboelectric potentials as gate signals to modulate semiconductor transport [3]. In both cases, the device can return to its initial state once the stimulus is removed, without necessarily retaining or processing stimulus history.

The transition from triboelectric sensing to sensory intelligence becomes meaningful when the generated signal participates in a defined information-processing operation. TENG-driven artificial synapses can convert mechanical stimulation into history-dependent synaptic responses [4], while tribotronic touch memories can preserve stimulus-dependent electrical states after the original contact has ceased [5]. Related neuromimetic tactile systems further demonstrate that spike timing, particularly first-spike timing, can encode rapidly changing information during touch and grasp [6,7]. Their significance therefore arises from the specific physical transformation imposed on the input and from the functional role of that transformation in the intended sensory task, extending beyond the emulation of biological synaptic or neuronal responses.

**Figure 1:**
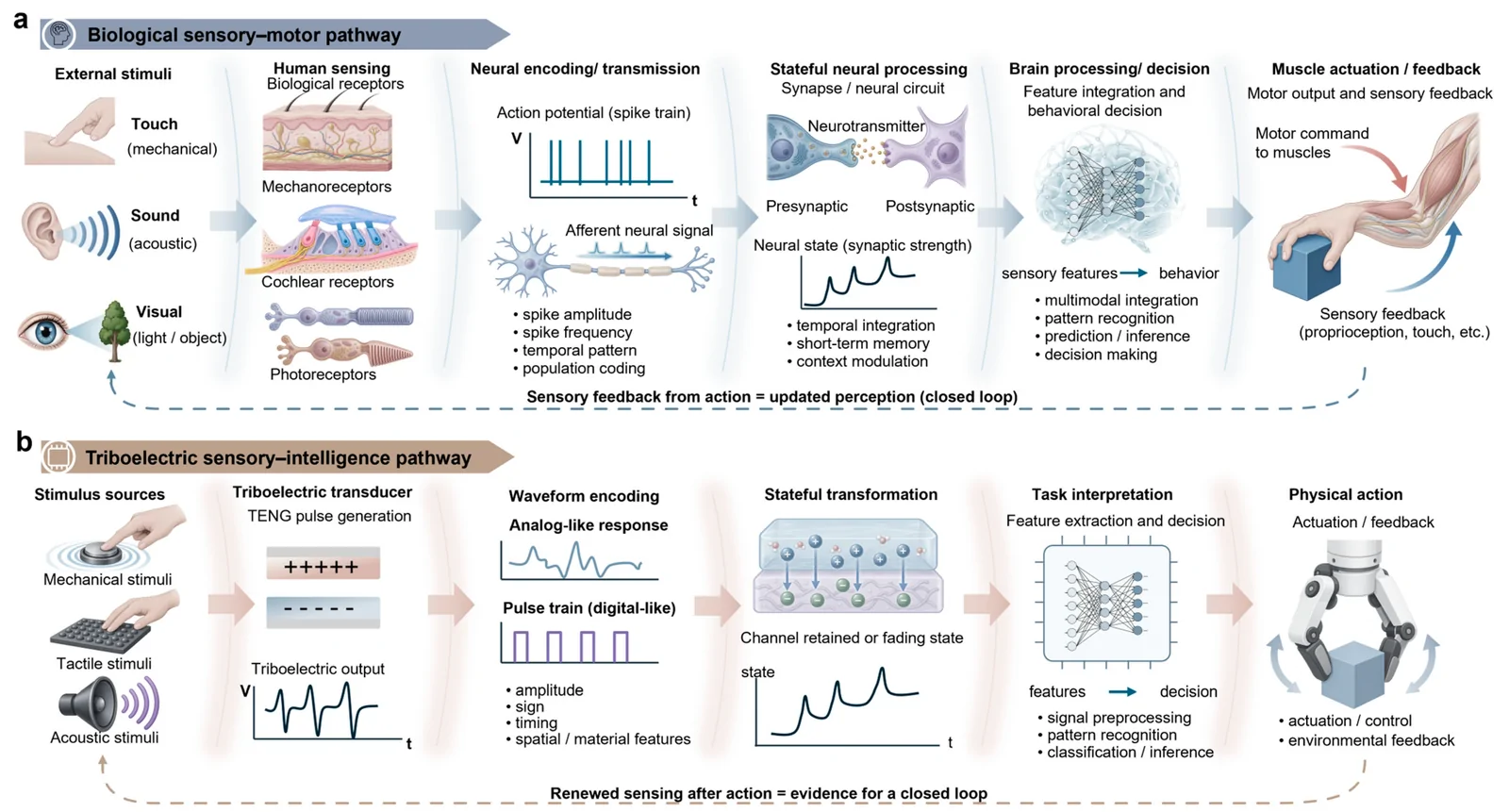
Functional correspondence between biological sensory–motor processing and triboelectric sensory intelligence. (**a**) In the biological pathway, external stimuli are detected by specialized receptors, encoded and transmitted as neural activity, modulated through state-dependent synaptic and neural processing, integrated by the brain for decision-making, and translated into motor output. Sensory feedback from the resulting action updates subsequent perception and closes the loop. (**b**) In the triboelectric sensory-intelligence pathway, mechanical or acoustic stimuli are converted into electrical outputs by triboelectric transducers, represented through waveform or pulse-based encoding, transformed by stateful devices, interpreted for task-relevant information, and coupled to physical action. Renewed sensing of the resulting interaction provides feedback for subsequent processing and control.

This information-centric perspective establishes a functional correspondence between biological sensory–motor processing and triboelectric sensory intelligence (Figure 1). In biological systems, external stimuli are detected by specialized receptors, encoded and transmitted as neural activity, modulated through state-dependent synaptic and neural dynamics, integrated by the brain for feature extraction and decision-making, and translated into motor commands. The sensory consequences of the resulting action are subsequently fed back to update ongoing perception (Figure 1a). A corresponding sequence of physical operations can be identified in triboelectric sensory-intelligence systems: mechanical and acoustic stimuli are converted by triboelectric transducers into electrical outputs, represented through waveform amplitude, polarity, timing, pulse sequences, or spatial and material-dependent features, transformed by stateful devices, interpreted for task-relevant information, and coupled to physical action. Renewed sensing after action allows the consequences of that action to influence subsequent processing and control, thereby completing the sensory–motor loop (Figure 1b). Recent reviews on triboelectric neuromorphic touch sensors, triboelectric–memristive coupling, and neuromorphic tactile transducers reflect the increasing convergence of sensing, memory, and neural-inspired processing in this field [8,9,10,11].

A key requirement of this framework is to distinguish information flow from energy flow. Triboelectric signals can convey sensory information, modulate device states or supply electrical energy, but these roles require different experimental evidence [1,11,12]. For example, triboelectric touch can write a memory state without external writing power, while subsequent electrical readout still requires a separate bias [5].

Recent reviews have examined self-powered memristive systems [11], integration between TENGs and field-effect transistors [12], self-powered neuromorphic touch sensors and triboelectric neuromorphic electronics [8,9,10,13,14,15], arrayed triboelectric tactile sensing [16], and artificial nerves for neuromorphic robotics and bioelectronics [17]. These reviews provide complementary accounts of device mechanisms, integration strategies and sensory applications. A remaining need is to connect these perspectives across the sensory pathway, relating the electrical conditions for state formation to the location of processing and the experimental evidence for task-level functionality. Against this background, this Review adopts an information-centric perspective on triboelectric sensory systems, tracing the progression of mechanical information from electrical encoding to state modulation, processing, interpretation, and feedback. Particular emphasis is placed on the coupling between triboelectric signals and physical state variables, including stored charge, polarization, ionic or electrochemical states, and resistive states, as well as on the boundary between functions implemented in physical hardware and those carried out by downstream software or simulation. Table 1 summarizes the scope of related reviews and the complementary focus of the present work.

The framework is organized along two complementary dimensions: the degree of coupling among sensing, state modulation, and computation, and the physical location at which each information transformation occurs. The processing-location dimension follows the established distinction among in-sensor, near-sensor and off-sensor computing [18], while structural integration describes how the transducer and state-bearing components are physically combined. In some architectures, triboelectric signals are delivered to separate local stateful devices; in others, sensing and state modulation share materials, interfaces, electrodes, or device structures. These dimensions are applied to representative demonstrations alongside quantitative coupling conditions, state lifetimes, implementation depth, validation protocols, and energy and latency scope. This comparison connects local device responses with the functions realized along the complete information pathway and identifies the remaining gaps toward scalable, adaptive and autonomous triboelectric sensory intelligence.

## 2. Triboelectric Sensing and Mechanical Information Encoding

Triboelectric sensing begins with the physical conversion of mechanical interaction into an electrical representation. The resulting voltage, current, transferred charge, polarity, and temporal structure are jointly determined by contact electrification, device geometry, mechanical trajectory, and electrical boundary conditions. For sensory applications, these outputs therefore serve as representations of mechanical interaction whose value depends on their stability, distinguishability, and relevance to the intended task. This section follows the formation of these representations from triboelectric transduction and interface engineering to temporal, spatial, material-specific, and multimodal encoding, and finally considers the separability and reliability of the resulting sensory representations.

### 2.1. Triboelectric Transduction of Mechanical Stimuli

**Figure 2:**
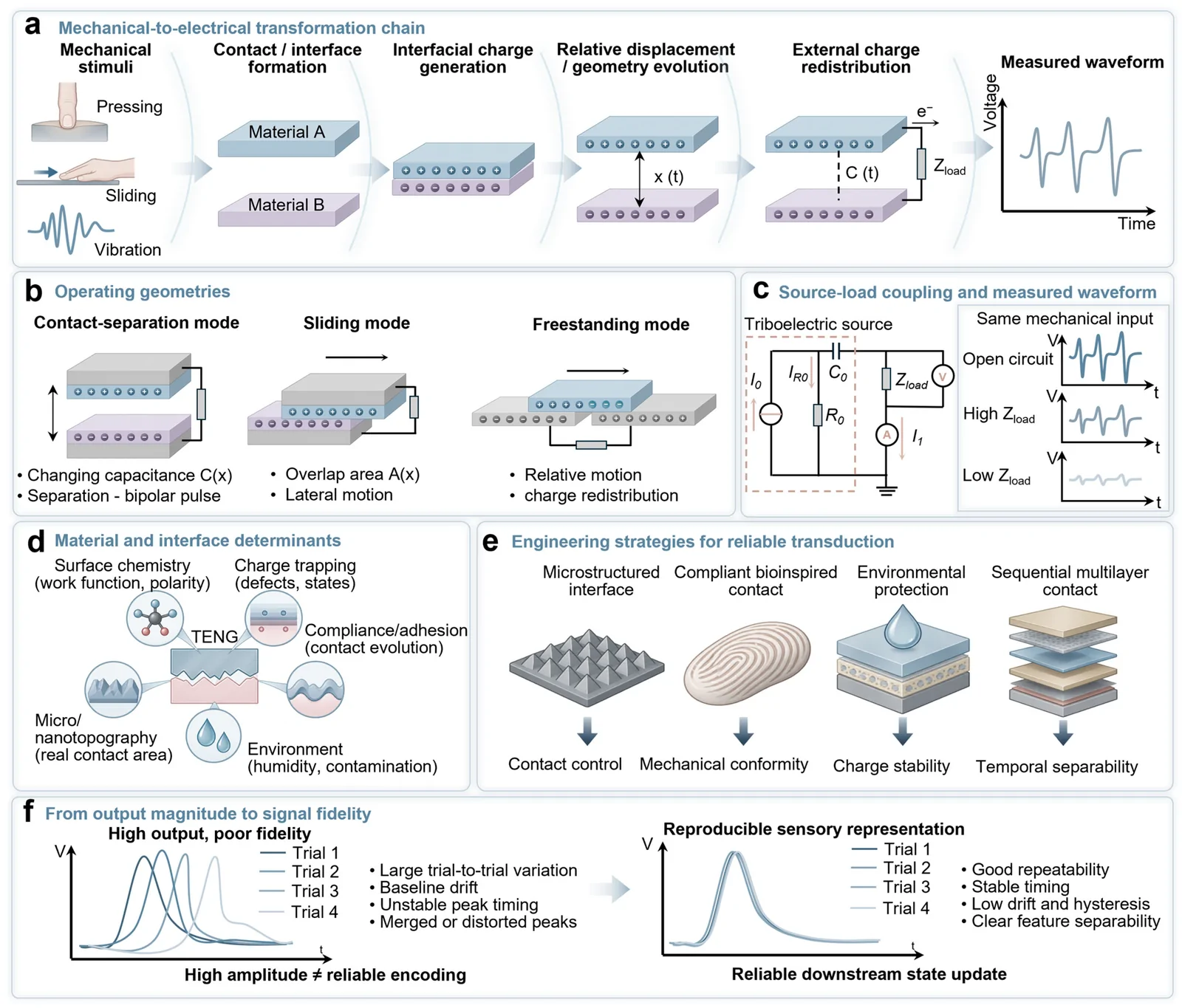
Physical transduction and signal fidelity in triboelectric sensing. (**a**) Mechanical-to-electrical transformation from external interaction to interfacial contact electrification, geometry-dependent charge redistribution and the resulting electrical waveform. (**b**) Representative operating geometries, including contact–separation, sliding and freestanding configurations, which produce distinct relationships between mechanical displacement, capacitance or overlap, charge transfer and electrical output. (**c**) Source–load coupling of a triboelectric transducer, illustrating how the measured waveform depends on both the mechanical input and the electrical boundary conditions imposed by the load or receiving device. (**d**) Material- and interface-level factors governing signal generation and stability, including surface chemistry, charge trapping, micro/nanotopography, compliance, adhesion and environmental conditions. (**e**) Representative engineering strategies for improving transduction reliability, including microstructured interfaces, compliant bioinspired contacts, environmental protection and sequential multilayer contact for enhanced temporal separability. (**f**) Conceptual comparison between high but variable electrical outputs and reproducible sensory representations, emphasizing that reliable encoding depends on repeatability, temporal stability, low drift and hysteresis, and clear feature separability rather than output magnitude alone.

A triboelectric waveform arises from the coupled effects of contact electrification, geometry-dependent electrostatics and electrical loading [1]. Contact establishes interfacial charge, whereas subsequent relative motion changes the electrostatic configuration and drives charge redistribution through the external circuit [1,19]. The measured voltage, current and transferred charge therefore reflect the complete transformation from mechanical interaction and interfacial charging to geometry-dependent charge redistribution, and depend on device geometry, displacement trajectory, motion rate and the electrical load (Figure 2a) [19,20]. Consequently, a peak voltage cannot generally be interpreted as a context-independent measure of force or pressure without specifying the mechanical configuration and electrical readout conditions.

The electromechanical transfer relationship also depends strongly on the operating geometry (Figure 2b). In contact–separation devices, mechanical displacement changes both the electrostatic driving potential and the effective capacitance, while the connected load governs the extent and rate of charge redistribution [19]. Sliding configurations instead vary the overlap between oppositely charged surfaces and thereby modulate lateral charge transfer [20]. Freestanding configurations use the motion of a charged element relative to stationary electrodes to generate electrical output without requiring the moving element to be electrically connected [21]. Rotational TENGs extend sliding configurations to angular motion, with segmented rotor–stator structures producing periodic changes in overlap and electrostatic induction. Angular position governs the charge-transfer cycle, while rotational speed and segment number influence the output frequency and current, enabling self-powered rotational-speed sensing [22]. These geometrical differences produce distinct relationships among displacement, transferred charge, current and voltage. Quantitative sensing therefore requires architecture- and load-specific calibration rather than a universal force-to-voltage conversion.

Device geometry can nevertheless be designed so that the mechanical–electrical relationship is more directly interpretable. In freestanding vibration sensors, for example, controlled motion of a charged element relative to fixed electrodes enables mechanical displacement to be inferred from the electrical response under defined operating conditions [21]. By contrast, in compliant contact sensors, the measured response can additionally depend on changes in contact configuration and interfacial mechanics, making the relationship between force and electrical output more device-specific. At the microscopic level, electron transfer provides one important framework for understanding contact electrification, although the microscopic origin of charge transfer remains dependent on the materials and interfaces involved [23]. Experimentally, triboelectric charge measurements are also sensitive to the selected material pair and measurement protocol [24].

At the circuit level, the TENG can be described to first order as a geometry-dependent capacitive source coupled to a finite electrical load (Figure 2c) [19]. Both the capacitance and the electrostatic driving term vary during mechanical motion, while the external impedance determines the loaded voltage, current and transferred charge observed at the receiving circuit. The electrical response should therefore be characterized under defined mechanical trajectories and load conditions that reflect the intended operation. Charge-limiting processes provide an additional constraint: air breakdown can restrict the experimentally accessible triboelectric charge density and thereby obscure the intrinsic charging capability of a material pair [25].

Compared with other mechanical-to-electrical transduction mechanisms, triboelectric sensing combines sensitivity to contact and motion with the ability to generate electrical excitation directly from mechanical events. Piezoelectric transducers also generate charge directly and are well suited to dynamic force and vibration sensing, although charge leakage limits sustained-load measurements [26]. Piezoresistive and capacitive sensors provide complementary access to static and slowly varying mechanical states, with electrical bias or excitation required for readout [27,28,29]. Electromagnetic transduction is effective for rotational and vibrational motion, but magnets and coils introduce size and mass, and the induced voltage decreases with slower magnetic-flux variation [30]. As summarized in Table 2, triboelectric transduction offers advantages for contact-triggered sensing and state modulation, while its finite charge supply, high source impedance and sensitivity to contact conditions make source–receiver matching central to reliable operation.

### 2.2. Materials and Device Engineering for Signal Fidelity

Materials and device structure determine not only the magnitude of triboelectric output, but also how reproducibly mechanical information is represented in the electrical waveform. Surface chemistry, charge retention, micro/nanotopography, compliance and environmental conditions jointly influence charge generation, persistence and contact evolution during loading and release (Figure 2d) [31,32]. Increasing signal amplitude can improve detectability, but this advantage is limited if contact mechanics, surface charge or environmental conditions introduce comparable variability. Signal fidelity therefore depends on the stability of the complete stimulus-to-waveform relationship rather than on peak output alone. Protocol-defined triboelectric-series measurements illustrate the importance of specifying the contacting pair and measurement conditions when comparing materials [24], while standardized charge-density measurements further show that experimentally accessible output can depend on the measurement configuration and charge-limiting processes [25]. Material rankings are therefore specific to the contact interface and operating conditions under which they are established.

Micro- and nanoscale structuring provides a direct route to modifying both electrical output and contact mechanics, forming one of several complementary strategies for improving signal fidelity (Figure 2e). Patterned surfaces can increase the effective contact area and alter the deformation, engagement and separation of the interface [33], while bioinspired structures can further tailor mechanical contact for tactile sensing [34]. The resulting enhancement may arise from several coupled effects including changes in compliance, local contact distribution and separation dynamics, which can influence both signal amplitude and waveform characteristics. Structural optimization should therefore consider the complete loading–unloading response together with cycle-to-cycle repeatability, hysteresis and temporal stability.

Surface and environmental engineering provide complementary strategies for stabilizing triboelectric signals through improved charge retention and environmental tolerance (Figure 2d,e). Hydrophobic and humidity-resistant structures can mitigate output degradation under humid conditions [35], whereas strategies that reduce triboelectric charge decay can improve the persistence of the generated charge state [32]. Surface-charge visualization and standardized quantification can further help distinguish genuine changes in charge distribution from variations introduced by the measurement procedure [31]. Signal stability should therefore be assessed through drift, repeatability and waveform preservation under representative operating conditions. In systems coupled to memory or neuromorphic devices, this requirement extends across the source–receiver interface, because variability in the receiving device can directly affect the reproducibility of state modulation.

Interface and structural design can further enhance the separability of mechanically encoded events. Multilayer triboelectric structures, for example, can convert sequential mechanical engagement into controllable electrical spike sequences [36]. Such behavior illustrates a broader design principle for sensory intelligence: the relevant objective is the preservation of stimulus-dependent features with sufficient repeatability, temporal stability and separability to support subsequent state modulation and interpretation (Figure 2f).

### 2.3. Amplitude, Polarity, and Temporal Encoding

**Figure 3:**
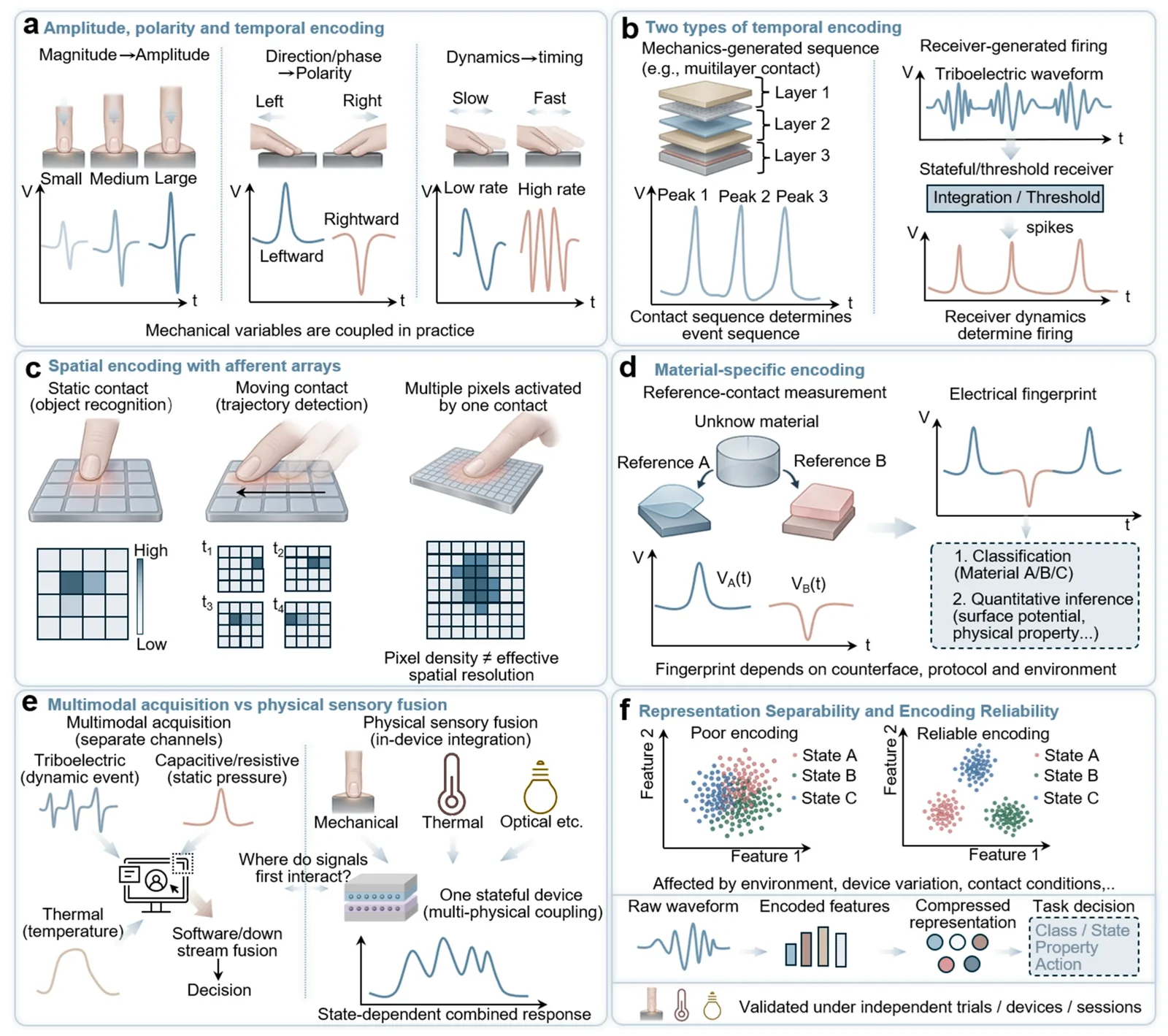
Triboelectric waveform encoding and sensory information representation. (**a**) Mechanical information can be represented through waveform amplitude, polarity and temporal structure, corresponding to stimulus magnitude, direction or phase, and dynamic features such as repetition rate or event timing. (**b**) Two routes to temporal encoding: mechanically generated event sequences arise from sequential contact interactions, whereas receiver-generated firing results from the dynamics of a nonlinear or thresholding stateful device driven by the triboelectric waveform. (**c**) Spatial encoding in distributed sensor arrays, illustrating static activation maps, temporally ordered recruitment during moving contact and the distinction between pixel density and effective spatial resolution. (**d**) Material-specific encoding using controlled reference contacts to generate electrical fingerprints that can support either categorical material recognition or quantitative inference of physical properties. (**e**) Multimodal sensing architectures, distinguishing separate acquisition and downstream fusion of complementary channels from physical multisensory integration in which multiple inputs interact within a common stateful element before readout. (**f**) Representation separability and encoding reliability, emphasizing the balance between within-state variability and between-state separability, the transformation from raw waveforms to encoded or compressed representations, and robustness against variations in environment, device characteristics and contact conditions.

Triboelectric signals can represent mechanical information through amplitude, polarity and temporal structure, providing complementary electrical representations of stimulus magnitude, motion direction or phase, and mechanical dynamics (Figure 3a). Under controlled conditions, signal amplitude can reflect the magnitude of displacement or mechanical interaction [21], whereas polarity can distinguish opposite phases or directions of motion in motion-dependent architectures [20]. Temporal features, including pulse number, spacing and order, can encode sequential or repeated contact events [36]. These signal dimensions are inherently coupled: changes in motion speed or contact trajectory can simultaneously alter peak current, pulse width and inter-event interval, while rectification can preserve event occurrence while suppressing directional information carried by signal polarity. The usefulness of signal conditioning should therefore be evaluated by the task-relevant information retained, modified or discarded during processing.

Temporal structure can originate directly from the mechanics of the triboelectric interface, with sequential contact events being mapped into corresponding electrical event sequences (Figure 3b). Multilayer TENGs, for example, can generate multiple spikes from a single touch through sequential contact and separation of spatially separated friction layers [36]. A complementary example is provided by triboelectric handwriting interfaces, in which spatially distributed vector sensors convert successive pen movements into direction-dependent pulse sequences, allowing the temporal order of sensor activation to represent the writing trajectory [37]. In such architectures, the number and timing of the resulting electrical events are defined by the physical contact sequence itself. This establishes a direct physical mapping from mechanical dynamics to temporally structured electrical representations. The fidelity of this encoding depends on reproducible contact mechanics, because changes in engagement, release or loading trajectory can shift spike timing or merge nominally distinct events. Temporal encoding therefore requires waveform reproducibility in addition to conventional sensitivity.

A distinct route to temporal encoding emerges when the triboelectric waveform drives a nonlinear or thresholding receiver, with output timing governed by the internal dynamics of the coupled device (Figure 3b). In triboelectric mechanoreceptor and auditory-neuron architectures, the TENG provides the sensory excitation, while a bistable device converts the incoming signal into discrete spikes [38,39]. The resulting firing behavior is jointly determined by the triboelectric waveform and the switching, integration and relaxation dynamics of the receiver. Input magnitude, polarity, device threshold and relaxation dynamics can therefore determine whether and when a spike is generated. Mechanically generated event sequences and receiver-generated firing thus represent two physically distinct forms of temporal encoding: the former originates in the contact sequence, whereas the latter emerges from the dynamics of the coupled transducer–receiver system.

Population timing extends temporal encoding from individual events to distributed sensory populations, in which the recruitment order of spatially separated sensing units can itself carry information about the evolving contact (Figure 3c). In neuromimetic tactile systems, different afferent units can become active at different stages of touch or grasp, allowing relative spike timing and recruitment order to encode dynamic tactile information [6]. First-spike timing can make discriminative information available early in the interaction, reducing the observation time required for subsequent classification [6]. Such timing-based representations are sensitive to event jitter, missed spikes and variations in mechanical trajectory, making the robustness of relative timing an important consideration alongside the latency advantage of early-event encoding. Triboelectric encoding can thus operate at several representational levels, from waveform amplitude and polarity to mechanically structured event sequences, receiver-generated spikes and population timing. These modes arise from different physical transformations and preserve different aspects of the underlying mechanical interaction. The choice of encoding strategy should therefore follow the physical variable of interest and the requirements of subsequent stateful processing or interpretation.

### 2.4. Spatial and Material-Specific Encoding

Spatial information can be represented through sensor identity, relative response magnitude and the temporal order in which distributed sensing sites are activated, forming static activation maps or dynamically evolving recruitment patterns (Figure 3c). In an array, a local mechanical event can therefore be encoded as either a spatial response pattern or a temporally ordered sequence of sensor activations during motion across the sensing surface [40,41]. A static response map primarily describes the location and spatial extent of contact, whereas a temporally ordered sequence can additionally contain information about direction, trajectory and deformation dynamics. Spatial encoding is therefore jointly determined by array geometry, the spatial extent of the mechanical interaction and the trajectory of the contacting object.

Array architecture determines the fidelity with which these spatial representations can be resolved. Tribotronic transistor arrays can convert localized contact into spatially distributed electrical responses [42], while graphene-based active matrices [43] and monolithically integrated indium gallium zinc oxide tribotronic arrays [44] provide alternative routes to combining sensing and addressable readout. Increasing the number or density of sensing elements improves spatial sampling, although nominal pixel density alone does not determine effective spatial resolution because a single physical contact can activate multiple neighboring sensing sites (Figure 3c). Mechanical spreading of the contact, crosstalk between neighboring sites and the dimensions of the contacting object can broaden the spatial response beyond the nominal device pitch. Spatial performance should therefore be assessed through experimentally resolvable features, localization accuracy and crosstalk under representative contact conditions together with the mechanical dimensions of the stimulus.

Material-specific encoding exploits the dependence of triboelectric output on the interacting material pair. Controlled reference contacts can therefore generate characteristic electrical fingerprints of an unknown material (Figure 3d). Triboelectric tactile systems, for example, use waveforms generated against a defined counterface as contact-dependent electrical representations for material recognition [45]. Such fingerprints can contain information in signal magnitude, polarity, temporal structure or combinations of waveform features, with their interpretation defined by the reference material and contact conditions under which they are acquired. The resulting representation can support categorical identification of known materials or, when appropriately calibrated, inference of an underlying physical property.

Reference-based sensing can extend this concept beyond categorical recognition, with the same contact-derived representation supporting either class assignment or quantitative inference depending on the downstream interpretation (Figure 3d). Dual-reference triboelectric measurements, for example, have been used together with regression models to predict surface potential directly from the measured electrical responses [46]. Multiple controlled contacts can provide complementary observations and reduce ambiguity associated with a single reference interface. These two tasks represent distinct levels of inference: classification assigns an unknown sample to a learned category, whereas quantitative prediction estimates an independently measurable material property. Material-specific encoding should therefore be evaluated with respect to the counterface, contact protocol and environmental conditions used to generate the signal, and quantitative predictions should, where possible, be validated against independently measured material properties [46].

### 2.5. Multimodal and Multiplexed Encoding

Multimodal sensing broadens the observable information available from an interaction by combining channels with complementary transfer characteristics, either as separately acquired signals or through their interaction within a common stateful element (Figure 3e). Its value arises from the complementary physical information provided by different transduction pathways and their ability to resolve ambiguities present in individual channels. Hybrid triboelectric–capacitive sensors, for example, can combine transient triboelectric responses with additional contact-dependent information for object recognition [29], while triboelectric devices have also been engineered to resolve pressure and temperature within the same sensing platform [47]. The effectiveness of multimodal encoding therefore depends on whether each channel contributes distinguishable information that remains robust against cross-sensitivity and environmental variation.

One important form of complementarity is the separation of rapidly varying and quasi-static mechanical information. Triboelectric channels are particularly responsive to changes in contact, sliding and vibration, whereas resistive, capacitive or related pressure-sensing mechanisms can retain information during sustained loading. Fingertip-inspired sensors combining triboelectric sliding detection with piezoresistive pressure sensing illustrate how dynamic surface interactions and normal force can be represented through separate physical channels [27]. Related hybrid tactile systems combine pressure- and vibration-sensitive responses [48], while fast- and slow-adapting channels have been integrated to emulate complementary temporal responses to mechanical stimulation [49]. These approaches extend the sensory representation across transient and sustained mechanical regimes, while preserving the physical origin of each signal.

Multimodal encoding can also combine channels that respond to different physical quantities. Multifunctional triboelectric architectures can extract several tactile features from structured interfaces [50], whereas hybrid thermoelectric–triboelectric platforms provide distinct electrical responses to thermal and mechanical inputs [51]. In the latter case, the signals can further converge on a common neuromorphic device, allowing multiple sensory inputs to influence a shared electronic state [51]. Such convergence represents a distinct level of integration because multiple sensory inputs jointly modulate a common physical state before readout. Temperature-dependent output, for example, may originate from a dedicated thermal transducer, from temperature-induced changes in contact mechanics or from changes in the triboelectric interface itself. Multimodal calibration should therefore preserve the physical origin of each observable feature while characterizing the principal cross-sensitivities between channels.

At the system level, synchronization, crosstalk, unequal signal quality and missing channels can compromise multimodal inference. Here physical fusion means that inputs interact in a common nonlinear or stateful element before readout; parallel acquisition followed by software fusion is a different architecture (Figure 3e).

### 2.6. Representation Separability and Encoding Reliability

Representation quality concerns how reliably stimulus states remain distinguishable relative to within-state variation under the intended operating conditions (Figure 3f). Channel count, analog-to-digital converter resolution and recognized class count are not measures of Shannon channel capacity or mutual information. No such information-theoretic quantity is estimated here. Instead, the comparison concerns separability, repeatability, drift and robustness to nuisance variation. Protocol-defined charge measurements [24] and reference-based surface-potential prediction [46] illustrate why the acquisition conditions and physical target must be specified before comparing an encoding.

Preserving more raw waveform samples is not necessarily equivalent to preserving more task-relevant information, because encoding and compression can reorganize the original waveform into lower-dimensional representations while retaining selected task-relevant features (Figure 3f). Distributed sensory representations can encode position or contact progression through the relative activity of multiple sensing units [52], whereas first-spike timing can compress a dynamic tactile interaction into an early temporal representation that becomes informative before a longer observation window is completed [6,53]. Such transformations can improve efficiency when they retain the variables required for the intended task, but they may also discard information that becomes relevant under different conditions. Early temporal coding, for example, can shorten the time required for interpretation while increasing sensitivity to timing variation or missed events. The value of an encoding strategy should therefore be assessed against an appropriate raw signal or less processed representation under matched mechanical conditions.

Encoding reliability depends on whether the representation remains separable when environment, device characteristics and contact conditions vary. Reference measurements can help isolate interface changes [46]. Statistical validation must additionally preserve the physical unit of independence, as detailed in Section 6.3; waveform windows from the same contact are not independent repetitions [54].

The practical objective is a sensory representation whose task-relevant distinctions survive the variations expected in deployment. Device-informed simulations and augmented samples can test a model, but they provide different evidence from new mechanical recordings. Their provenance and the partitioning of the original observations must remain traceable [54].

## 3. Coupling Triboelectric Signals with Memory and Computing Devices

### 3.1. Electrical Matching Between Triboelectric Sources and Stateful Devices

**Figure 4:**
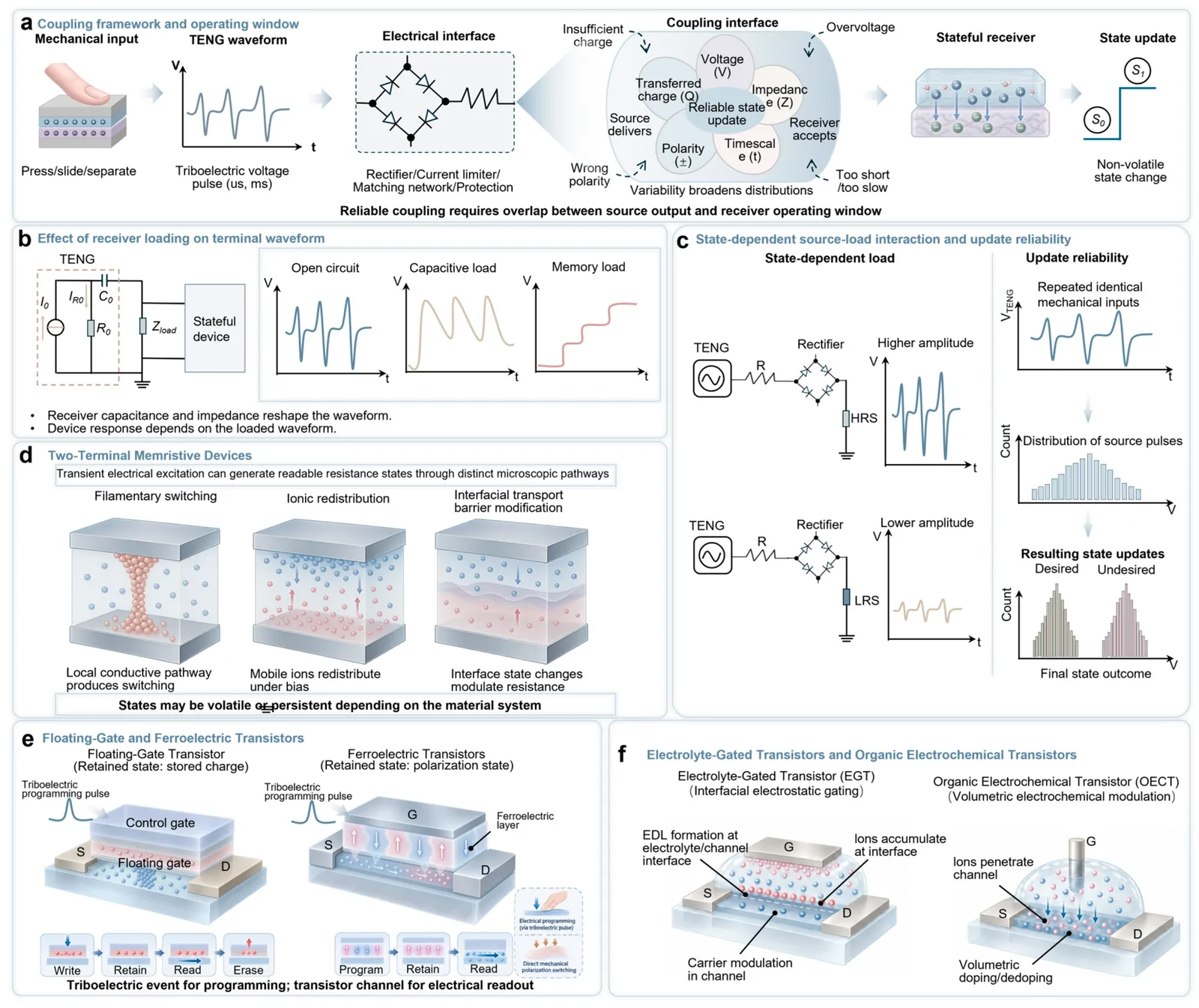
Coupling triboelectric signals to stateful electronic devices. (**a**) Coupling framework from mechanical stimulation and triboelectric waveform generation to the electrical interface, stateful receiver and resulting state update. Reliable programming requires overlap between the source output and receiver operating window in terms of transferred charge, voltage, impedance, polarity and characteristic timescale. (**b**) Effect of receiver loading on the terminal waveform. Receiver capacitance and impedance reshape the signal delivered by the TENG, producing different responses under open-circuit, capacitive-load and memory-load conditions. (**c**) State-dependent source–load interaction and update reliability. Changes in receiver resistance alter the loaded waveform, while pulse-to-pulse variability broadens the distribution of programming conditions and can produce both desired and undesired final states. (**d**) Representative two-terminal memristive mechanisms, including conductive-filament formation and rupture, ionic redistribution and interfacial transport-barrier modification, which convert transient electrical excitation into volatile or persistent resistance states. (**e**) Floating-gate and ferroelectric transistors as longer-lived stateful platforms. Floating-gate devices retain information through stored charge and follow a write–retain–read–erase sequence, whereas ferroelectric transistors retain information through polarization states that modulate channel transport; in both cases, a triboelectric event can provide the programming stimulus while the transistor channel provides electrical readout. (**f**) Electrolyte-gated transistors and organic electrochemical transistors (OECTs), highlighting the distinction between interfacial electric-double-layer gating and volumetric electrochemical modulation.

Coupling a triboelectric source to a stateful device requires the electrical output of the transducer to overlap with the operating window of the receiver. Under load, the delivered voltage must reach the relevant programming range, sufficient charge must be transferred to modify the internal state, and the source and receiver impedances must permit this transfer on a compatible timescale. Signal polarity can additionally determine the direction of state evolution. These parameters jointly define a source–receiver coupling window within which a mechanical event can reproducibly generate the intended state transition (Figure 4a). Table 3 compares reported source outputs and receiver operating conditions, distinguishing open-circuit measurements, loaded pulses and electrical programming tests.

The receiving device is part of the electrical load and can substantially reshape the waveform delivered to its terminals. The time-varying capacitance of a TENG, together with load resistance and parasitic capacitance, governs the amount and rate of charge transferred to the connected circuit [55]. A simple charge-sharing estimate illustrates this constraint. At a fixed mechanical configuration, connecting a source capacitance C_T_, initially at its open-circuit voltage V_oc_, to an initially uncharged receiver capacitance C_R_ gives V_R_ ≈ V_oc_C_T_/(C_T_ + C_R_). Here, C_R_ includes the effective receiver, interconnect and measurement capacitances. Reaching a programming voltage V_prog_ therefore requires C_R_/C_T_ ≤ V_oc_/V_prog_ − 1. For an illustrative source with C_T_ = 100 pF and V_oc_ = 10 V, reaching a 1 V programming target requires C_R_ ≤ 900 pF; a 1 nF receiver reaches approximately 0.91 V. These assumed values illustrate charge sharing rather than represent the literature measurements. Although the estimate neglects continuing mechanical motion, leakage and nonlinear device dynamics, it explains why a high unloaded voltage does not guarantee effective programming.

In the triboelectric biristor mechanoreceptor and its subsequent array implementation, mechanically generated charge accumulates until regenerative switching occurs, while the connected load influences the delivered current [39,56]. A quantitative example of the charge constraint is provided by the contact-electrification-activated transistor study: an approximately 9 V TENG event transfers less than 3 nC, whereas the resulting transistor current corresponds to only about 0.5 V of conventional gate drive [57]. The study reports an input capacitance of approximately 900 pF and a total gate charge of approximately 10 nC at V_gs_ = 4.5 V and V_ds_ = 15 V [57]. The TENG charge is thus less than 30% of that gate-charge value, indicating a charge deficit for the specified operating point. However, bias dependence and Miller feedback prevent treating the input capacitance as a fixed divider element, and the 0.5 V value represents current-equivalent gate control rather than a directly measured divider voltage. These results demonstrate that effective gate modulation depends on the charge delivered under the coupled source–receiver condition rather than on the unloaded voltage alone.

Coupling can also evolve as the receiver changes state. In tribotronic resistive memories, transitions between high- and low-resistance states change the effective load presented to the triboelectric source, thereby altering voltage division and the waveform subsequently delivered to the device [58]. Rectifying elements and array architectures can further constrain current paths and suppress unintended transitions [59]. A nominally identical mechanical input can therefore produce different programming conditions as the source waveform and receiver state vary (Figure 4c). For example, a rectified and loaded TENG circuit delivers an approximately 4.5 V pulse lasting about 40 ms under a 1 MΩ external load [59].

**Table 3:** Reported source outputs and receiver programming conditions.

Study and Configuration	Source Output and Conditions	Receiver Programming and State Response	Coupling Characteristics
Yu et al. [57], TENG-driven transistor	≈9 V; transferred charge < 3 nC per event.	C_iss_ ≈ 900 pF; total gate charge ≈10 nC at V_gs_ = 4.5 V and V_ds_ = 15 V; current-equivalent gate drive ≈ 0.5 V.	Charge-limited gate modulation with substantial Miller-related attenuation.
Kim et al. [59], Al/FEP TENG + rectifier + N:TaO_x_ memory	Conditioned pulse: ≈4.5 V, ≈40 ms with a 1 MΩ external load.	Eight resistance levels observed over 500 s; separate retention > 10^4^ s after 3 V programming.	Rectification and resistive loading provide a mechanically driven programming pulse.
Fang et al. [60], arch TENG + ferroelectric transistor	Open-circuit output: ≈220 V under finger tapping; duration above 100 V: ≈2.7 ms.	Electrical gate sweep: −60 to +40 V at V_ds_ = −8 V; TENG-written on/off states.	High-voltage triboelectric output enables ferroelectric state programming.
Kim et al. [61], polymer TENG + ferroelectric synapse	Touch: 1.86–2.44 V, ≈12 ms; release: negative pulse, ≈50 ms.	Electrical update pulses: ±1 V, 20 ms; tested amplitudes 0.1–1 V and durations 5–20 ms.	Volt-scale mechanical pulses drive synaptic modulation in a ferroelectric receiver.
Sultan et al. [62], ionogel tactile OECT	Contact-dependent output: ≈0.05–0.9 V; ≈0.5 V for PET at 5.6 kPa.	Electrical gate pulses: 1 V, 1–10 s at V_ds_ = −0.5 V.	Contact-dependent triboelectric gating modulates an ionogel-based electrochemical channel.
Cho et al. [63], flexible triboelectric-gated ion-gel transistor	Positive peaks: 0.6–3.0 V at 0.25–2.0 N; negative peak magnitudes: 1.01–5.3 V.	Electrical pulses: ±0.5–1.5 V, 10–100 ms; fitted EPSC decay times: ≈70–450 ms, depending on stimulation.	Triboelectric gating produces ionic responses with stimulation-dependent relaxation.

Different state mechanisms impose distinct electrical requirements for successful programming. Resistive switching depends strongly on programming voltage and compliance current; charge-storage devices depend on transferred charge; ionic and electrochemical devices depend on both delivered charge and its timescale; and regenerative switching elements require sufficient accumulated excitation to reach a firing threshold. These differences make source–receiver matching specific to the intended state transition: voltage amplitude must be considered together with charge availability, pulse duration and receiver dynamics. Reliable coupling ultimately depends on whether the loaded triboelectric source can reproducibly induce the intended state update across different initial states and stimulation histories.

Receiver programming is governed by the state mechanism and stimulation protocol. Ferroelectric synaptic weight updates have been demonstrated using ±1 V, 20 ms electrical pulses, whereas the coupled triboelectric source generates touch pulses lasting approximately 12 ms [61]. This difference highlights the importance of pulse duration and delivered charge when translating electrical characterization into mechanical programming. In ion-gel transistors, electrical pulses of ±0.5–1.5 V and 10–100 ms produce fitted EPSC decay times of approximately 70–450 ms, with longer memory exceeding 2 s under different stimulation histories [63]. Ferroelectric transistors exhibit polarization-dependent state modulation over a −60 to +40 V gate sweep, although the sweep range does not directly specify the switching threshold [60]. These studies show that source–receiver compatibility depends on both the programming stimulus and the resulting state dynamics. Table 3 summarizes these operating conditions.

### 3.2. Two-Terminal Memristive Devices

In two-terminal memristive devices, mechanical excitation can be converted into a stimulus-dependent resistance state that either persists after stimulation or relaxes over a characteristic timescale. The underlying state variable may involve the formation and rupture of a conductive filament, ionic redistribution, or the evolution of an interfacial transport barrier (Figure 4d). For triboelectric operation, the relevant issue is the reproducibility with which the mechanically generated electrical waveform establishes or modifies this internal transport state under realistic circuit conditions, including series impedance, compliance constraints, rectification, and reset requirements. A resistance change observed under externally applied electrical pulses therefore characterizes the intrinsic switching behavior of the device, whereas mechanically driven operation additionally requires direct coupling between the triboelectric source and the state transition.

Recent studies extend this picture beyond filamentary switching. In tribotronic resistive memories, state-dependent source–load interaction allows mechanical stimuli to be recorded as resistance changes [58], while self-rectifying oxide memristor crossbars demonstrate mechanically written spatial resistance patterns within addressed arrays [59]. These examples illustrate that two-terminal architectures can share a common sensory function while relying on substantially different microscopic switching mechanisms. Filamentary and interface-type devices may differ in switching stochasticity, compliance requirements, rectification behavior, and susceptibility to electrical disturbance, yet both can convert transient mechanical excitation into a subsequently readable resistance state.

The interpretation of self-powered operation also depends on the complete write–reset–read pathway. In the N:TaO_x_ system, single-cell pressure-driven switching is demonstrated together with an externally applied electrical reset, whereas the array architecture employs an appropriate connection scheme to enable TENG-driven SET and RESET operations [59]. Subsequent retrieval of the programmed resistance pattern still requires an applied read voltage. Such demonstrations establish mechanically addressable memory and spatial state programming, while the broader computational function depends on how writing, readout, reset, and subsequent information processing are physically integrated. Diffusive Ag memristors provide a complementary reference for volatile switching and spontaneous relaxation [64], although electrically driven implementations should be interpreted primarily as mechanistic references unless direct triboelectric coupling is experimentally demonstrated.

Filament-engineered hexagonal boron nitride (h-BN) devices provide a further example of threshold-responsive resistive dynamics coupled to triboelectric excitation. The reported mechano-nociceptor architecture links a TENG input to Ag-filament-mediated switching, illustrating how mechanical stimulation can access a threshold-dependent resistive state [65]. The computational significance of such dynamics depends on the extent to which sensing, state transformation, and downstream processing are experimentally connected within the same physical pathway. Reservoir-computing demonstrations based on intrinsic device dynamics therefore represent a separate level of implementation when the triboelectric sensory input is not directly incorporated into the demonstrated computing operation [65]. Quantitative comparisons of switching windows, reset requirements, and end-to-end energy should likewise be based on measurements performed across the completely coupled system.

### 3.3. Floating-Gate and Ferroelectric Transistors

Charge-storage and ferroelectric transistors provide persistent state variables through distinct physical storage mechanisms (Figure 4e). Floating-gate and charge-trapping architectures confine carriers in an electrically isolated or localized charge reservoir, whereas ferroelectric devices retain state through the orientation or magnitude of polarization. In both cases, a triboelectric event can provide the programming stimulus while the transistor channel provides a separate electrical readout [66,67,68,69]. These architectures therefore convert a transient mechanical event into an electronic state that remains accessible after the original stimulus has ended.

In charge-storage devices, retention and rewritability depend on where charge is confined and, on the barriers, controlling its injection and removal. Tribotronic floating-gate transistors and van der Waals heterostructures have demonstrated mechanically programmable nonvolatile and multilevel states [66,67,68,69]. Tunneling barriers, trap distributions and erase conditions determine the efficiency of state programming, the stability of the retained charge and the reversibility of subsequent updates. Device operation is therefore defined by the complete write–retain–read–erase cycle, encompassing programming efficiency, retention, readout and reset or erase behavior.

Ferroelectric devices use the triboelectrically generated programming potential to establish or modify a polarization state that subsequently modulates channel transport [60,61,70,71]. The mechanical-to-state conversion therefore proceeds through two coupled steps: mechanical interaction generates the triboelectric electrical stimulus, which then modifies the ferroelectric polarization. Direct mechanically induced polarization switching follows a different conversion pathway, in which mechanical stress acts directly on the ferroelectric material [72]. The location of this conversion is important for distinguishing triboelectrically mediated electrical programming from direct electromechanical switching. Charge storage and ferroelectric polarization can persist well beyond the duration of the original mechanical event, providing long-lived state variables with retention and rewrite characteristics distinct from rapidly relaxing ionic or electrochemical states.

### 3.4. Electrolyte-Gated Transistors and Organic Electrochemical Transistors

Electrolyte-gated devices introduce state through the redistribution of mobile ions. Triboelectric excitation can drive ionic accumulation or migration, thereby establishing an electrostatic or electrochemical state that can persist after the mechanical excitation has decayed [73,74]. The lifetime of this state is governed by ion transport, interfacial capacitance, material chemistry and device geometry, providing a mechanism for extending the temporal influence of a transient mechanical input.

The underlying state mechanism depends strongly on device architecture. In electric-double-layer and related electrolyte-gated transistors, ions predominantly redistribute near the semiconductor–electrolyte interface, where the resulting interfacial electric field modulates the channel carrier density; triboiontronic MoS_2_ and proton-conductor transistors provide representative examples [75,76]. In organic electrochemical transistors, ions can penetrate the active channel and modulate its bulk redox or doping state, as demonstrated in ionogel-coupled OECT sensory synapses [62]. These two regimes therefore differ in both the location and nature of state formation: interfacial ionic accumulation primarily produces electrostatic modulation, whereas volumetric ion penetration produces electrochemical modulation throughout the active channel (Figure 4f). The resulting differences in ion-accessible volume, transport length and material interactions give rise to distinct response and relaxation dynamics.

A residual ionic state also creates history dependence between successive mechanical inputs. When the interval between inputs is comparable to the device relaxation time, the ionic distribution established by one event can alter the response to the next [74,77,78,79]. At longer intervals, the ionic distribution progressively approaches its baseline condition and the influence of the preceding event diminishes. The ratio between the input interval and the characteristic relaxation time therefore determines the degree of temporal interaction between successive stimuli.

Across the device classes discussed above, the central distinction is the physical state variable and its characteristic lifetime. Volatile resistive states and interfacial ionic accumulation can relax over relatively short timescales; volumetric electrochemical modulation can extend state evolution through ion transport and retention within an active channel; and trapped charge, ferroelectric polarization and persistent resistive switching can maintain states over substantially longer intervals. These mechanisms therefore span a continuum of state lifetimes and physical storage modes that can be matched to different stages of sensory processing.

## 4. Architectures and Functions of Triboelectric Sensory Computing

### 4.1. Integration Architectures and Processing Location

**Figure 5:**
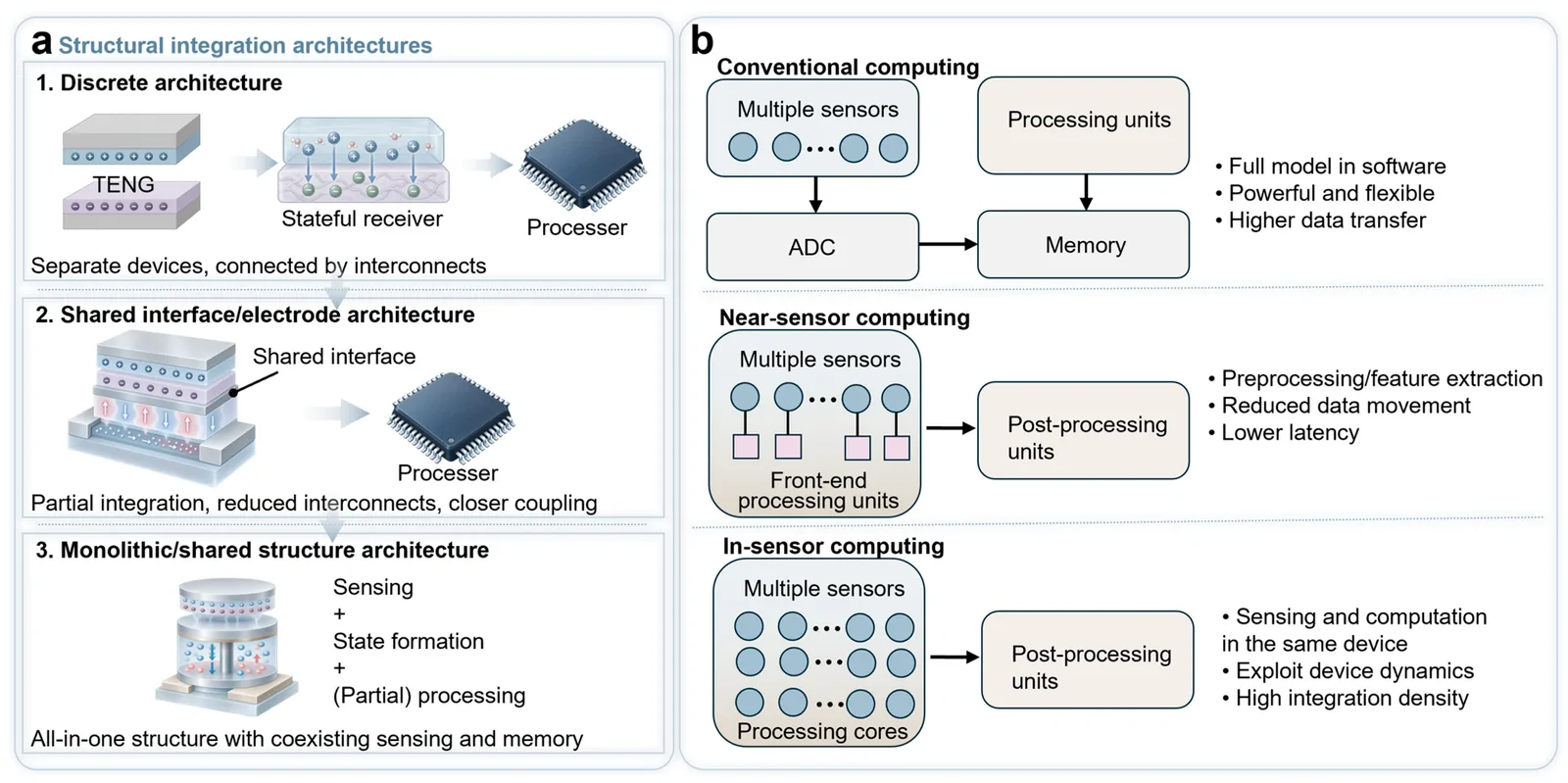
Structural integration and processing location in triboelectric sensory computing. (**a**) Structural integration from discrete transducer–receiver–processor architectures to shared-interface/electrode and monolithic structures with increasingly localized sensing and state formation. (**b**) Processing location from conventional off-sensor computing to near-sensor and in-sensor computing. Structural integration and computation location represent complementary, rather than equivalent, dimensions of system design. Panel (**b**) is redrawn from Ref. [18].

Once a triboelectric signal can reproducibly establish a physical state, sensory-computing systems differ in where transduction, state formation and retention, readout, and information processing are physically implemented. These functions may remain in discrete components, share interfaces or electrodes, or coexist within a common device structure. Triboelectric sensory-computing architectures can therefore be described along two complementary dimensions: the degree of structural integration and the physical location of information processing (Figure 5). The first describes how closely sensing and state-forming elements are physically coupled, whereas the second identifies where useful transformations of the sensory representation occur.

Structural integration spans discrete, shared-interface/electrode and monolithic or shared-structure architectures (Figure 5a). In a discrete architecture, the TENG, stateful receiver and processor remain separate components connected through external interconnects. Shared-interface or shared-electrode architectures partially integrate these functions by allowing a common physical interface to participate in both transduction and state modulation, reducing interconnect requirements and strengthening source–receiver coupling. At a higher level of structural integration, the triboelectric interface, dielectric, electrolyte or active layer can participate directly in sensing and state formation within a common device structure, enabling sensing and memory—and in some cases partial processing—to coexist within the same physical platform [80]. Structural integration therefore describes the physical organization and coupling of these operations; the extent of computation is determined separately by the information transformation performed within the integrated structure.

Processing location provides the second architectural dimension (Figure 5b). In conventional or off-sensor computing, triboelectric sensors primarily generate electrical representations that are digitized and transferred to external memory and processing units. Near-sensor architectures position front-end operations such as signal conditioning, feature extraction or stateful preprocessing close to the sensing array, reducing data movement and latency [18,81]. In-sensor computing moves the transformation further into the sensing hardware, such that device dynamics or local processing elements directly modify, integrate or extract information from the sensory input before downstream readout. A discrete stateful receiver can therefore perform local temporal processing without monolithic integration, whereas a structurally integrated sensor can remain limited to transduction and storage. Structural integration and processing location consequently form complementary coordinates for comparing system architectures.

Triboiontronic systems provide a representative example of how structural integration can be realized through an active ionic interface. Electric-double-layer modulation, proton conduction and related ionic processes can allow the contact or electrolyte interface to participate directly in both signal generation and state evolution [75,76,82,83,84]. When the same ionic interface contributes to transduction and subsequent state modulation, it functions as part of a shared sensing–state pathway; when it receives an already generated triboelectric signal, it instead acts as a downstream stateful element.

### 4.2. Sensory Memory and History-Dependent Processing

Sensory memory emerges when a state generated by an earlier mechanical interaction remains accessible and influences a subsequent sensory operation. At the functional level, the key questions are where the retained state resides, what information it preserves and how long it remains available. Short-lived states can retain recent-event context, whereas more persistent states can preserve accumulated stimulus history over longer intervals.

Memory can reside at different positions along the sensory pathway. In some devices, previous stimulation modifies the transducer itself, causing a subsequent mechanical event to generate a different electrical response [85]. In others, the triboelectric signal writes a separate electronic state that can later be read independently of the original mechanical input [86]. Shared sensory–memory structures place transduction and state retention within a common functional element. These configurations therefore represent different locations of memory within the information pathway: an upstream retained state modifies subsequent transduction, whereas a downstream state preserves a representation after transduction has occurred.

The form of retained information can likewise vary. A persistent conductance state can encode accumulated stimulation, while a spatial resistance pattern can preserve the locations at which mechanical programming occurred [59]. Sensory-memory transistors can instead allow prior excitation to modulate the response to a subsequent electrical or mechanical input [77]. Sensory memory is therefore defined by the preservation of an identifiable feature of previous stimulation and its accessibility to a subsequent operation.

A useful description is the write–state–read relationship. The write event establishes a physical state, the state persists over a characteristic interval, and the subsequent read operation determines whether the stored history influences later sensing or processing. This framework also helps distinguish functional sensory memory from incidental history dependence arising from surface-charge decay, mechanical relaxation or environmental drift. The retained state becomes computationally relevant only when its dependence on previous stimulation is subsequently used to transform or interpret sensory information.

Tribotronic MoS_2_ touch memory illustrates sensory-state retention within a shared sensing–memory structure. Mechanical contact charges the PDMS layer, modifying the transistor electrostatics and producing a threshold shift of up to approximately 3.5 V that persists for almost 1 h [5]. The retained interfacial charge stores the contact history without an externally supplied electrical writing pulse, while subsequent electrical readout converts this state into a measurable channel response. Memory is therefore established before readout through the same interface that receives the mechanical stimulus. This architecture provides persistent sensory storage, with retention and repeated operation governed by the stability and neutralization of the stored charge.

A complementary approach couples a separate TENG to an interface-type N:TaO_x_ resistive-memory array through a rectifier and external resistor [59]. Circuit conditioning delivers an approximately 4.5 V, 40 ms programming pulse, enabling eight resistance levels observed over 500 s and spatial-pattern storage in a 6 × 6 crossbar. Intrinsic rectification suppresses unwanted current paths and supports array operation. Here, mechanical information is transferred from the transducer into resistance states in a separate memory structure, extending sensory storage from a single channel to a spatial array. Together, these examples distinguish memory based on retained triboelectric charge from memory written into a resistive receiver, while illustrating the additional readout, reset and addressing operations associated with array-based storage.

### 4.3. Synaptic Dynamics and Temporal Processing

**Figure 6:**
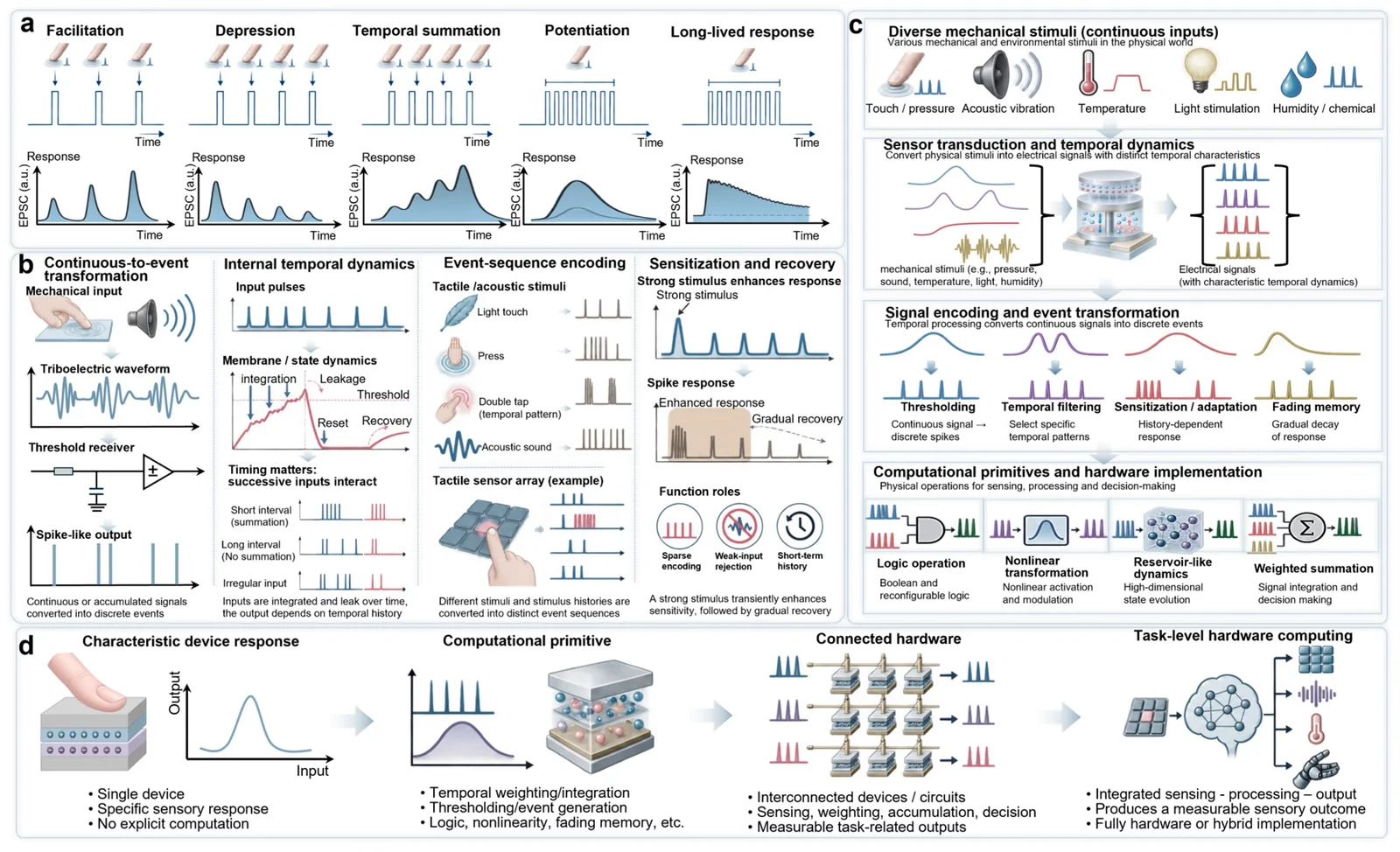
Physical processing functions and implementation levels in triboelectric sensory computing. (**a**) History-dependent synaptic dynamics, including facilitation, depression, temporal summation, potentiation and longer-lived modulation of response strength. (**b**) Threshold and event-based transformation, in which continuous or accumulated sensory excitation is converted into discrete events through integration, thresholding, reset and recovery dynamics. (**c**) Representative computational primitives, including state-dependent logic, temporal filtering, nonlinear fading-memory transformation and weighted summation. (**d**) Increasing depth of physical implementation from characteristic device responses and computational primitives to connected hardware and task-level sensory computing.

Synaptic dynamics describe history-dependent transformations in which the response to a current mechanical event is conditioned by the state established by preceding stimulation. Representative behaviors include facilitation, depression, temporal summation, potentiation and longer-lived modulation of response strength (Figure 6a) [61,66,74]. Their functional significance lies in the temporal weighting, integration and retention of sensory events.

Short-term plasticity provides a direct route to temporal selectivity. When successive mechanical inputs arrive before the internal state has fully relaxed, residual charge, ionic redistribution or other transient states can modify the response to subsequent events. Closely spaced inputs can therefore produce facilitation, depression or temporal summation depending on pulse interval, repetition frequency and the characteristic relaxation time of the device [4,74,78]. Otherwise similar mechanical events can consequently generate different outputs according to their recent temporal context.

More persistent state evolution extends this history dependence over longer timescales. Charge-storage, ferroelectric and mechanoplastic transistor systems can retain conductance changes after repeated stimulation and thereby encode accumulated event history or programmable weights [61,63,66,87]. Rapidly relaxing states are suited to transient temporal weighting and recent-event processing, whereas longer-lived states can support persistent adaptation or weight storage.

Synaptic-like dynamics and learning represent different functional levels. Facilitation, depression or persistent conductance modulation demonstrate state-dependent plasticity, while learning additionally requires those state changes to be used within a defined objective-driven update process. The computational relevance of synaptic dynamics therefore depends on the information-processing operation implemented with the evolving device state and on whether that operation contributes to a measurable task-level outcome.

### 4.4. Threshold and Event-Based Transformation

Event-based transformation converts continuous or accumulated sensory excitation into discrete electrical events through integration, thresholding and recovery dynamics (Figure 6b). In triboelectric mechanoreceptor and auditory-neuron systems, mechanically generated inputs can drive regenerative or bistable elements toward a switching threshold, thereby transforming an analog triboelectric waveform into a spike-like output [38,39]. This transformation provides a sparse event representation before subsequent processing.

The resulting event sequence is determined by the complete temporal dynamics of the receiver. Integration and leakage regulate the accumulation of successive inputs, threshold crossing triggers event generation, while reset and recovery determine the readiness and sensitivity to subsequent inputs. When inputs arrive before the internal state has fully relaxed, their effects can accumulate toward threshold; after firing, recovery dynamics influence whether and when another event can occur. Physical mechanoreceptor arrays and triboelectric neuron systems have used these mechanisms to convert tactile or acoustic inputs into temporally structured spike trains [38,56,88]. Event generation therefore transforms stimulus history into a sequence whose timing reflects both the sensory input and the internal dynamics of the receiver.

Related nonlinear state evolution can produce sensitization and recovery. Mechanically activated h-BN memristive systems, for example, exhibit threshold activation followed by altered responses to subsequent stimulation [65]. Similar threshold-dependent dynamics have been explored using diffusive memristors and other mechanically coupled neuromorphic devices [89,90,91,92]. Such responses can enhance sensitivity after strong stimulation and subsequently recover toward the baseline state over a characteristic timescale. Threshold and event-generating elements therefore occupy an intermediate functional level between transduction and higher-order computation. They can sparsify sensory signals, reject weak inputs, encode stimulus timing and retain short-term history through their internal dynamics. These operations provide useful event-based representations for subsequent temporal filtering, integration and decision-making.

### 4.5. Computational Primitives

Computational primitives are defined physical input–state–output transformations that select, combine or reorganize sensory information before higher-level interpretation (Figure 6c). The temporal weighting and event-generation processes described above already constitute such operations. Additional primitives include Boolean or state-dependent logic, temporal filtering, nonlinear fading-memory transformation and weighted summation. Their computational significance is determined by the transformation physically performed on the sensory input.

Logic provides a clear example because the input states and output rule can be explicitly defined. Tribotronic circuits have demonstrated Boolean operations controlled by triboelectric gating [93,94], while charge-trapping and reconfigurable transistor structures extend these concepts toward programmable or logic-in-memory behavior [87,95]. Such systems perform physical computation when the intended logical transformation is executed by the device or circuit itself.

Temporal filtering exploits state evolution to selectively transmit, suppress or emphasize temporal patterns. Ionic, electrochemical or capacitive states can respond differently to pulse interval, frequency or accumulated excitation [78,96]. When these dynamics are deliberately used to favor specific temporal features, the device functions as a physical temporal filter rather than simply exhibiting a time-dependent response.

Nonlinear fading-memory dynamics provide another useful computational primitive. Diffusive and other stateful memristive systems can map temporally varying inputs into nonlinear internal states that retain information about recent stimulation [64,65]. These dynamics constitute a reservoir substrate when the input is mapped into accessible internal states. Reservoir computing additionally requires those states to be sampled and combined through a trained readout. Nonlinear fading memory therefore describes a device-level computational resource, whereas reservoir computing describes a system-level implementation constructed from that resource.

Weighted summation provides the complementary operation of combining multiple inputs. Conductance states, transistor currents or parallel device branches can physically encode weights, while current or charge summation integrates contributions from multiple sensory channels. Together with temporal filtering, nonlinear transformation and logic, such operations form a compact set of physical primitives from which more complex sensory-computing functions can be assembled.

A connected auditory system illustrates how triboelectric encoding can be combined with physical computational primitives. A triboelectric auditory sensor supplies signals to biristor neurons, which perform integration and threshold-triggered spike generation, followed by charge-trap MOSFET synapses that weight the spikes [38]. The two-input, two-output architecture uses reference frequencies of 118 and 174 Hz and demonstrates discrimination between piano notes C3 at 130.8 Hz and G3 at 196.0 Hz through comparison of output frequencies. Its contribution lies in connecting sensory encoding, spike generation and synaptic weighting within a hardware pathway, with pitch information expressed in the weighted outputs. The limited pitch set and preset connection strengths define the scope of this demonstration: it establishes a connected processing primitive for pitch discrimination, while broader auditory recognition, adaptive learning and array scalability remain open challenges.

### 4.6. From Device Functions to Hardware Sensory Computing

The transition from device dynamics to sensory computing can be described by the depth of physical implementation (Figure 6d). At the first level, a device exhibits a characteristic input–output response. At the next, its internal state dynamics implement a defined computational primitive. Connected hardware then combines multiple physical operations, while task-level hardware computing requires the integrated system to produce a measurable sensory outcome. These levels describe how far computation is physically instantiated along the sensory pathway.

This distinction is particularly important for neuromorphic sensory demonstrations. A mechanoreceptor that transforms touch into spikes establishes physical event encoding. A software network constructed from measured device characteristics can then evaluate the computational utility of those responses, but the network operation itself remains external to the physical sensory hardware [56]. Device characterization, software-assisted evaluation and physically connected computation therefore represent different implementation levels.

More integrated demonstrations physically connect sensing to weighting, accumulation or decision elements. Triboelectric auditory systems, for example, have combined sensing, spike generation and synaptic weighting within a connected hardware network to generate task-related outputs [38]. At larger scales, physical afferent arrays can provide sensory representations while subsequent network layers remain software implemented [6,56]. These hybrid architectures provide an important intermediate step between isolated device primitives and fully physical computing systems.

A complete hardware sensory-computing pathway requires the relevant transformations to operate together. Mechanical interaction is first converted into an electrical representation; stateful devices then provide memory, temporal weighting or programmable conductance; multiple signals may be accumulated; nonlinear elements transform the combined response; and a physical readout produces a decision, classification, state estimate or control signal. Integrated memristive neural networks and in-memory-computing platforms provide broader reference points for the circuit-level coordination of these operations [97,98].

For triboelectric sensory computing, the central question is therefore the extent to which the pathway from mechanical interaction to information transformation and task-level output remains physically instantiated. This perspective separates characteristic device behavior from computational primitives, connected hardware and complete sensory-computing systems.

Table 4 compares representative triboelectric sensory computing demonstrations involving different state mechanisms and system architectures. L1 denotes a measured state response; L2 a defined physical primitive; L3 connected hardware performing multiple operations; and L4 a task-related decision or control output produced by the connected hardware pathway. H marks hybrid software-assisted interpretation. The label follows the demonstrated pathway. A software classifier running on a physical computer remains downstream software for this architectural comparison.

The comparison reveals a concentration of convincing local state formation and temporal/event primitives, with frequent software-assisted task evaluation. Structural sharing does not determine implementation depth: the discrete auditory system [38] reaches connected weighted outputs for a narrow task, whereas a shared tactile synapse can still rely on software classification [61,62,63]. Conversely, an addressed memory pattern [59] is stronger evidence of connected storage than a single hysteresis curve but does not demonstrate a classifier. In the glove-based system, acquired triboelectric signals are processed by a downstream CNN for gesture recognition [100]. The first-spike system [6] and mechanoreceptor array [56] establish physical encoding plus external network evaluation.

The non-triboelectric feedback system [28] illustrates how local state dynamics can support closed-loop tactile control and provides a reference for developing TENG-based sensing-to-action architectures. Across the triboelectric demonstrations compared in Table 4, mechanically induced state modulation and local preprocessing are more extensively demonstrated than adaptive control with complete system-level energy accounting. Further integration will require characterization of receiver loading across operating states, coordinated array readout and reset, validation under independent test conditions, and energy measurements over repeated sensing–processing–actuation cycles. Together, these advances would help translate device-level functionality into scalable and energy-autonomous sensory systems.

## 5. Intelligent Perception and Closed-Loop Interaction

The functional value of triboelectric sensory computing ultimately depends on the information that can be extracted from physical interactions and used to guide interpretation or action. At this level, the emphasis shifts from individual state mechanisms and computational primitives to task-relevant perception, including tactile and material inference, gesture interpretation, multimodal integration, embodied interfaces and sensory-driven control.

### 5.1. Tactile, Texture, and Object Perception

**Figure 7:**
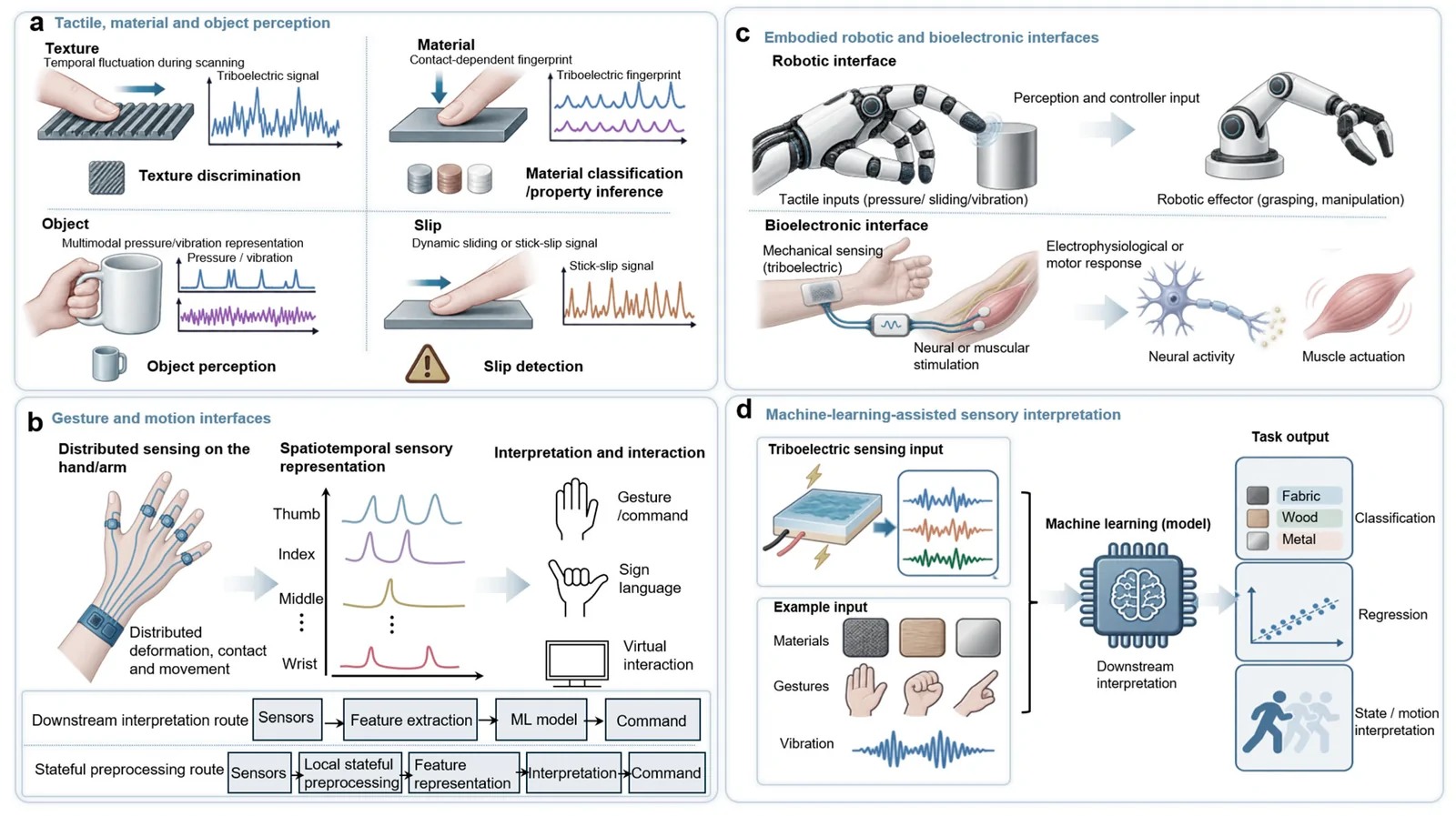
Task-level perception and interpretation in triboelectric sensory systems. (**a**) Distinct sensory representations for texture discrimination, material classification or property inference, object perception and slip detection. (**b**) Gesture and motion interfaces based on distributed spatiotemporal sensing, with downstream interpretation or local stateful preprocessing before command generation. (**c**) Robotic and bioelectronic interfaces linking mechanically encoded sensory information to robotic or biological effectors. (**d**) Machine-learning-assisted interpretation of triboelectric sensory representations for classification, regression and state or motion inference.

Tactile perception encompasses distinct inference targets, including texture, material identity or properties, object state and slip, each of which is represented by different features of the mechanical interaction (Figure 7a). Surface texture is commonly encoded through temporal fluctuations generated during sliding or scanning [101,102], whereas contact-dependent triboelectric responses can provide material-specific fingerprints for recognition or quantitative surface characterization [45,46]. Object perception can additionally benefit from complementary pressure, vibration or capacitive channels that capture different aspects of contact state [29,48]. Slip is characterized by dynamic sliding or stick–slip signatures associated with relative motion at the interface [27,102]. These tasks therefore correspond to distinct mappings from physical interaction to electrical representation and subsequent inference.

The sensing strategy should consequently be matched to the target physical variable. Triboelectric fingerprints emphasize the electrical interaction between a sample and a defined counterface [24], scanning interfaces transform surface topography and frictional interaction into temporally structured signals [101,102], and hybrid pressure–vibration architectures separate slowly varying contact forces from faster surface-induced fluctuations [48]. Material classification assigns an observation to a learned category, whereas quantitative property inference estimates an independently measurable physical variable and therefore requires appropriate calibration of the counterface, contact force, scanning trajectory and other relevant conditions [46].

Robust tactile perception further depends on the stability of these representations across variations in mechanical interaction. Texture and slip features obtained during controlled scanning can vary with normal force, sliding velocity, contact area and interface geometry. Perceptual performance should therefore be evaluated through both separability and invariance across the range of contact conditions expected during operation. This requirement links the physical fidelity of the triboelectric representation to the reliability of subsequent object, material or slip inference.

### 5.2. Gesture and Motion Interfaces

Gesture and motion interfaces transform distributed deformation, contact and movement into spatiotemporal sensory representations that can subsequently be mapped to gestures, language or control commands (Figure 7b). Wearable triboelectric sensors can encode hand and body motion through the relative activity and timing of multiple sensing sites [99]. The resulting representation depends jointly on local sensor response, sensor placement and the kinematics of the wearable structure, allowing distributed mechanical events to be interpreted as coordinated motion patterns.

The transformation from physical motion to semantic meaning can occur at different positions along the information pathway. In a conventional route, triboelectric sensors generate the sensory representation, feature extraction organizes the measured signals, and a downstream machine-learning model performs classification or command mapping [99]. Stateful preprocessing introduces an additional physical transformation before downstream interpretation, allowing temporal context, event history or local feature selection to be incorporated within sensory hardware (Figure 7b) [100,103].

Such preprocessing is particularly useful when the meaning of a gesture depends on temporal order or recent interaction history. Residual device states can weigh successive events differently, while complementary contact or proximity information can help separate intentional gestures from incidental motion [103]. The functional contribution of local stateful processing therefore lies in modifying the representation presented to the downstream interpreter, for example, through temporal selection, history-dependent weighting or suppression of ambiguous events.

### 5.3. Robotic and Bioelectronic Sensory Interfaces

Embodied robotic and bioelectronic interfaces extend triboelectric perception from externally imposed measurements toward sensory pathways in which the encoded information can influence a robotic or biological effector (Figure 7c). Distributed triboelectric sensors can map deformation, contact and motion across robotic structures [40], while tactile scanning systems can reconstruct surface information during controlled interaction [101]. Hybrid fingertips and electronic skins further combine pressure, bending, sliding or vibration information to provide richer representations of contact state [27,34]. At this level, sensory performance is determined by the usefulness of the encoded information for subsequent robotic or biological response.

For robotic interfaces, mechanical integration places additional requirements on the stimulus-to-signal mapping. Repeated deformation, impact and surface contact can alter sensor geometry and electrical characteristics. Stretchable and self-healable tribotronic devices provide routes to recovering electrical functionality after deformation or damage [104]. Functional recovery, however, also requires reconstructed sensor response to preserve the mechanical information needed for perception or control.

Bioelectronic interfaces provide a complementary pathway in which mechanically generated sensory signals influence neural or muscular activity. Triboelectric sensory receptors and neural-stimulating electronic skins have been used to couple mechanical interaction to biological responses [105,106], while organic artificial afferent nerves and artificial neural tactile systems provide broader reference architectures for transmitting tactile information toward biological or robotic effectors [107,108]. Electrophysiological activation, evoked movement and task-level sensory restoration represent distinct functional endpoints and should therefore be identified explicitly when evaluating these interfaces.

### 5.4. Multimodal Perception and Sensory Fusion

Building on the encoding architectures introduced in Section 2.5 and Figure 3e, multimodal perception combines complementary sensory responses to resolve ambiguities in individual channels. Triboelectric–capacitive sensing combines transient contact signals with additional contact-dependent information, while mechanical–thermal sensing extends perception to distinct physical quantities [29,51]. The contribution of each modality should be assessed against the corresponding single-modality response under comparable conditions.

Shared-state devices provide a further route to multimodal processing. In mechano-photonic and multimodal synaptic devices, mechanical, optical or other inputs modulate a common electronic, ionic or polarization state [70,71,109,110]. Their responses can therefore depend on both the combination and timing of the inputs, enabling sensory history and context to influence subsequent readout. These studies demonstrate physical interactions between modalities, although task-level interpretation may still require downstream processing.

The examples above highlight complementary roles for multimodal sensing: independent channels broaden the observable features of an interaction, whereas shared-state devices introduce context-dependent transformations before readout. Their perceptual benefit can be evaluated through comparisons of recognition accuracy, robustness or ambiguity reduction using individual and combined inputs. For shared-state devices, varying the relative timing and strength of the inputs can further establish how their physical interaction contributes to the measured response.

### 5.5. Machine-Learning-Assisted Sensory Interpretation

Machine learning provides a flexible route from triboelectric sensory representations to higher-level inference (Figure 7d). Depending on the task, measured signals can be used for categorical classification, continuous-property regression or interpretation of motion and physiological states. Reference-based triboelectric measurements, for example, can be combined with regression models to estimate continuous material properties [46], while tactile and vibration signals can be classified using downstream learning models [56,111]. Wearable interfaces similarly map multidimensional mechanical signals to gestures or rehabilitation-related states [30,99].

These tasks probe different forms of sensory information. Material recognition assigns observations to predefined categories [45], surface-potential estimation predicts a continuous physical variable [46], gesture recognition interprets spatial and temporal motion patterns [99,100,112], and vibration identification operates on temporally structured mechanical signals [111]. Accuracy values obtained across these tasks therefore describe different inference problems and should be interpreted within their respective input distributions, targets and evaluation protocols.

The physical location of information processing provides a second distinction. Some architectures use triboelectric sensors primarily as an input stage, with feature extraction and inference performed in software [99,100]. Others introduce physical spike encoding or local stateful preprocessing before software-based task evaluation [56,61,62,63]. Machine learning should therefore be located explicitly within the information pathway by identifying which transformations occur physically before the representation reaches the algorithm. For deployment, generalization depends on whether task-relevant features remain informative across changes in user, device, session, object and contact condition. At the perception level, the key requirement is that the learning model operates on a sensory representation whose physical variability and domain of validity have been adequately characterized.

The evaluation protocols differ in the physical unit over which generalization is assessed. Among the six studies summarized in Table 5, participant-grouped validation is reported in one study, whereas device- and session-held-out evaluation are not explicitly described in the examined material. Material holdout and tests on novel word combinations address complementary forms of generalization. These differences place reported performance within distinct evaluation settings and highlight the importance of preserving the relevant physical unit of independence during data partitioning.

### 5.6. Closed-Loop Sensory–Motor Interaction

Closed-loop sensory–motor interaction extends perception into action by allowing the consequences of a sensory-driven response to influence subsequent sensing. A complete loop therefore couples sensing, interpretation and actuation through a return pathway in which the action changes the physical interaction and the resulting state is subsequently sensed again, distinguishing closed-loop regulation from a purely feed-forward sensory–motor chain.

Triboelectric systems have demonstrated several components of this pathway. Mechanically generated signals can drive neural stimulation, biological responses or robotic effectors, establishing physical links from sensing toward action [113]. Related tactile and neural-interface systems further demonstrate transmission of sensory information toward motor or robotic outputs [6,107,108]. These sensing-to-action pathways become closed-loop systems when the resulting action modifies the physical state and renewed sensing is used to determine the subsequent response.

Robotic tactile control provides a useful functional reference. A piezoresistive tactile sensor coupled to an organic electrochemical synaptic transistor provides a non-triboelectric example of tactile feedback in programmed robotic control [28]. In a robotic manipulation loop, for example, slip detection can trigger an adjustment in grip force, after which renewed tactile sensing determines whether stable contact has been restored or further correction is required. Closed-loop performance can therefore be evaluated through quantities such as correction time, disturbance rejection, grasp stability and residual control error, depending on the application.

Closed-loop interaction represents the point at which triboelectric sensory intelligence becomes an ongoing sensory–motor process. Mechanical events are encoded, interpreted and translated into physical action, while the consequences of that action generate the next sensory input. Renewed sensing after action is therefore the defining experimental evidence that closes the sensory–motor loop. Although individual sensing, processing and actuation functions have been demonstrated, their integration into stable, adaptive and repeatedly operating triboelectric feedback systems remains a major challenge.

## 6. Evaluation Framework for Triboelectric Sensory Intelligence

### 6.1. Evaluation Across the Sensory Information Chain

Evaluation of triboelectric sensory intelligence should follow the successive transformations of information along the sensory pathway. The relevant metrics therefore depend on the stage being evaluated, progressing from representation fidelity and state-update reliability to physical processing and task-level generalization. At the transduction stage, performance is determined by the reproducibility, stability and separability of the mechanical-to-electrical representation. At the source–receiver interface, the relevant quantities are the magnitude, variability and reproducibility of the resulting state update. At the processing stage, evaluation concerns whether the intended physical transformation, such as temporal filtering, thresholding, accumulation or nonlinear mapping, is performed consistently on the encoded input. Task-level perception introduces a further requirement: the resulting representation and processing pathway must remain informative beyond the specific mechanical trials used for calibration or training. These quantities characterize different stages of the information pathway and together provide a system-level description of sensory performance.

The appropriate metric consequently depends on the function being demonstrated. Standardized TENG figures of merit provide valuable measures of electrical generation under defined mechanical and electrical conditions [114], whereas sensory applications additionally require characterization of the information represented by the generated waveform. Likewise, conductance modulation, retention and relaxation describe the state dynamics of a memory or neuromorphic device, while classification or regression performance reflects the behavior of a larger sensing–processing–inference pipeline. A meaningful evaluation should therefore identify the correspondence between mechanical input, electrical representation, internal state transformation and final observable output.

Variability can propagate and change form as information moves through this pathway. Variations in contact mechanics modify the source waveform; source variability is subsequently transformed by the receiver into a distribution of state updates; and downstream processing operates on those evolving states. Physical mechanoreceptor arrays and triboelectrically driven stateful devices illustrate how variability introduced at the source–receiver interface can influence the resulting sensory representation [56,73]. Characterizing variability at each transformation stage, together with its propagation through the connected pathway, therefore provides a more informative basis for system comparison than isolated component metrics.

### 6.2. Energy and Latency Across the Sensory Pathway

Energy consumption in triboelectric sensory systems is distributed across transduction, state modulation, readout, computation and actuation. Triboelectric excitation can supply the electrical stimulus for state formation, reducing reliance on an external programming source. This benefit is particularly relevant to event-driven architectures, in which mechanical inputs directly trigger charge accumulation, switching or synaptic modulation. Other stages, including channel readout and downstream processing, may retain separate electrical supplies. The overall energy balance therefore encompasses both mechanically supplied energy and electrical consumption across the connected sensory pathway [115,116].

The energetic benefits of triboelectric coupling take different forms across device architectures (Table 6). Contact-electrification-activated afferents combine triboelectric stimulation with low-voltage synaptic operation, achieving a reported local dissipation of 11.9 fJ/spike at a drain bias of 1 mV, excluding back-end processing and LED circuitry [73]. In resistive-memory arrays, rectification and loading convert the triboelectric output into approximately 4.5 V, 40 ms programming pulses, enabling mechanically driven state writing [59]. These demonstrations illustrate complementary routes to reducing externally supplied programming energy: low-voltage channel modulation and direct mechanical writing of persistent states. Their system-level benefit also depends on the energy associated with reading, selection, reset and subsequent computation.

Temporal performance reflects the interaction between mechanical acquisition, electrical encoding and receiver dynamics. First-spike encoding provides a means of extracting information before a complete mechanical waveform has been acquired. A tactile spiking system demonstrates 1.2 ms encoding resolution and reaches a defined minimum accuracy of 62.5% for curvature and stiffness within 0.32 s under the selected touch protocol [6]. The distinction between these timescales reflects the progression from precise event encoding to the accumulation of sufficient evidence for classification. Electrochemical receivers introduce another temporal contribution through ion redistribution and channel modulation. An organic electrochemical synaptic transistor exhibits an approximately 234 ms rise time and 12 ms recovery under the specified electrical test conditions [28]. These asymmetric dynamics shape the response to successive inputs and contribute to the temporal behavior of the connected sensory pathway.

Energy and latency are consequently linked through the location and timescale of information processing. Local state formation can retain or transform sensory information before readout, potentially reducing downstream sampling and computation. Persistent states support deferred retrieval but introduce read and reset operations, whereas relaxing states provide temporal processing whose usefulness depends on the relationship between decay time and stimulus interval. In closed-loop systems, these device-level properties interact with inference and actuation to determine the energy and delay of a completed response. The resulting tradeoff is therefore governed by how effectively each architecture converts mechanical events into task-relevant information across the full sensory pathway.

### 6.3. Reproducibility and Generalization

Reproducible comparison begins with a clearly defined mechanical and electrical experiment. For triboelectric transduction, contacting materials, contact geometry, mechanical trajectory, force or displacement conditions, environmental state and measurement configuration can all influence the observed signal. Standardized contact and charge measurements are therefore important for interpreting differences among devices [24,25]. When a stateful receiver is coupled to the triboelectric source, its initial state, programming history, delivered waveform, readout condition, relaxation behavior and reset protocol become part of the same experimental description.

The physical unit of independence is equally important when sensory signals are analyzed statistically. Multiple waveform windows, extracted features or augmented samples derived from a single mechanical trial remain observations of the same underlying physical realization. Training, validation and test partitions should therefore preserve the appropriate independent unit, such as contact trial, device, specimen, recording session or participant, according to the intended application. In machine-learning studies, distributing correlated samples from the same realization across different partitions can introduce information leakage and inflate apparent generalization performance [54]. Partitioning should consequently occur at the physical level before windowing, feature extraction or augmentation whenever these operations generate correlated derivatives of the same experiment.

Data provenance should remain traceable throughout the analysis pipeline. Measured physical observations, segmented or transformed records, augmented samples and model-generated data represent different levels of evidence. Data augmentation can increase the diversity of training inputs, while independent physical measurements provide the basis for evaluating robustness and generalization. This distinction is particularly important in sensory-computing studies that combine measured device responses with software networks, fitted models or device-informed simulations [56]. Reporting should therefore identify which stages are physically measured, which are derived from experimental data and which are implemented computationally.

Reproducibility also requires evaluation across the sources of variation relevant to deployment. Depending on the application, these may include repeated contacts, multiple devices, fabrication batches, specimens, users, recording sessions, environmental conditions or mechanical trajectories. Robust performance is established when the task-relevant representation and resulting output remain reliable across the variations expected within the intended operating domain. Taken together, reproducible evaluation requires traceability from physical stimulus to electrical representation, state evolution, computational processing and final output. Clear reporting of experimental conditions, independence structure, data provenance and implementation boundaries allows different triboelectric sensory architectures to be compared according to the functions that are physically demonstrated and the conditions under which those functions remain reliable.

## 7. Challenges and Perspectives

### 7.1. Reliable Sensing and Signal–Device Co-Design

A central challenge for triboelectric sensory intelligence is to preserve task-relevant information as mechanical interaction is transformed into an electrical representation and subsequently into a physical device state. Progress will therefore depend on establishing stable, interpretable and reproducible stimulus-to-state relationships, with electrical amplitude serving as one component of a broader information representation. Triboelectric output remains sensitive to the contacting pair, measurement protocol, surface-charge distribution and environmental conditions [24,25,31], while quantitative material sensing further illustrates how variability introduced at the contact interface can propagate into the inferred physical quantity [46]. Future materials, surface structures and device architectures should therefore be optimized for information fidelity, separability and stability across representative operating conditions. Reference channels and periodic calibration offer complementary routes to tracking contact and environmental drift, although their effectiveness depends on distinguishing nuisance variations from the mechanical information of interest.

The source–receiver interface constitutes a second reliability bottleneck. A stateful device responds to the loaded waveform delivered at its terminals, while available charge, source impedance, polarity, receiver state and programming history jointly determine the resulting state transition. Triboelectrically driven synaptic, resistive-memory and artificial-afferent systems already illustrate several forms of such coupled behavior [56,57,58,59,73]. The approximately 9 V, <3 nC triboelectric event associated with only about 0.5 V of current-equivalent gate control illustrates how finite charge availability can constrain receiver modulation despite a high source voltage [57]. These demonstrations establish local state modulation, while reproducible coupling across changing contact conditions and receiver states remains a constraint on system integration. Future development should increasingly treat the mechanical interface, triboelectric source, electrical coupling network and stateful receiver as a co-designed information pathway. Practical priorities include reducing interconnect and feedback capacitance, matching receiver charge requirements to the available triboelectric output, and conditioning pulse polarity and duration for the intended state transition. Such co-design can align source waveform characteristics with device thresholds, charge requirements and relaxation dynamics, thereby improving the reproducibility of state updates across repeated operation.

Continuous perception introduces an additional challenge because different aspects of mechanical interaction occupy different temporal regimes. Triboelectric channels are naturally sensitive to changes in contact, sliding and vibration, whereas capacitive, resistive and related mechanisms can retain quasi-static information during sustained loading [27,29,49,117,118]. Stateful electronics can introduce an additional timescale by retaining recent interaction history [81,119]. The resulting opportunity is to construct multiscale sensory representations that simultaneously capture rapid events, sustained contact and recent context. Robotic contact and slip monitoring provides a concrete application: a transient triboelectric channel can detect changes in interaction, while a complementary channel tracks sustained loading. Achieving this objective requires the instantaneous mechanical state to remain distinguishable from residual device states generated by preceding stimulation. A key future direction is therefore the controlled separation and integration of stimulus dynamics and device memory across multiple timescales. Matching receiver relaxation and reset to the task timescale can help preserve this distinction while retaining useful temporal context.

### 7.2. Scalable and Adaptive Sensory Computing

Scaling sensory intelligence requires more than increasing pixel count. As arrays expand, device variation, crosstalk, parasitic capacitance, addressing and reset pathways become coupled to the local state dynamics used for processing. Tribotronic transistor arrays and integrated tactile systems demonstrate the feasibility of spatially distributed sensing [42,44], while sensory-neuromorphic arrays increasingly extend physical processing across multiple sensing units [56,80,119]. These developments establish progress beyond isolated devices, although task-level perception often retains downstream software. The central scaling problem is therefore to maintain predictable information transformation as sensory computing expands across space, devices and interconnects. Selective addressing, local readout and controlled reset offer routes to limiting unwanted interactions and coordinating repeated state updates across the array. Their benefit depends on whether the added peripheral circuitry preserves the energy and latency advantages of local processing.

Manufacturing adds a second dimension of scalability. Large-area textile processing, printing and related additive approaches provide established routes for producing distributed triboelectric transducers [120,121]. Integrating stateful electronics introduces additional constraints associated with processing temperature, solvent compatibility, interface quality, alignment, encapsulation and interconnect density. Scalable fabrication should therefore be developed at the level of the complete sensing–state–computing stack. Modular sensory-computing tiles, heterogeneous integration and architectures tolerant to device variability may provide practical routes toward large-area implementation without requiring identical characteristics from every element.

Adaptation addresses scalability in time rather than space. Wearable and interactive systems inevitably encounter changes in users, contact conditions and device characteristics, but compensation for drift and acquisition of new behavior represent different forms of adaptation. Gesture interfaces and rehabilitation-oriented sensors already highlight the need to operate across variable users and conditions [30,99], while neuromorphic sensorimotor systems demonstrate how state updates can be coupled to changes in embodied behavior [122]. Future systems should distinguish at least two forms of adaptation: recalibration that restores a desired input–output relationship, and learning that modifies the sensory or control policy in response to experience. Learning becomes particularly meaningful when an explicit error, reward or feedback signal drives a physical state update that improves subsequent task performance. For wearable interfaces, periodic recalibration offers a practical near-term response to user and contact variability. Hardware learning introduces the additional requirement of linking feedback to controllable state updates, with the resulting improvement evaluated across repeated tasks and independent recording sessions.

### 7.3. Toward Autonomous Closed-Loop Sensory Systems

Autonomous sensory systems require coordinated sensing, stateful processing, feedback control and energy supply. A central design opportunity is to co-optimize waveform conditioning, source–receiver matching and local processing so that task-relevant sensory information is preserved throughout repeated operation.

Closed-loop operation requires action to modify the physical interaction and renewed sensing to guide subsequent responses. Organic artificial afferent systems provide a non-triboelectric reference for tactile feedback in robotic behavior [28], while triboelectric brain–behavior interfaces provide pathways from mechanical sensing toward action [113]. Building on these approaches, future TENG-based systems can incorporate renewed sensing into the control policy: a disturbance triggers an action, its physical consequences are measured, and subsequent responses are updated accordingly. Event-triggered acquisition, local state retention, and coordinated readout and reset offer potential routes to reducing continuous operation while maintaining the information needed for subsequent control. Integrating this repeated feedback with stateful processing and sustained energy supply is a key step toward autonomous sensory–motor systems.

Intermittent event-driven sensing and mechanically written memory offer plausible near-term applications because they exploit functions already demonstrated at the device and circuit levels. These applications align with sparse mechanical inputs: individual events can trigger a response or leave a retrievable state without continuous acquisition. Robotic contact and slip feedback offers a related opportunity where rapid detection can support corrective action, although controller and actuator energy remain part of the system budget. Continuously adaptive, energy-autonomous sensory–motor systems remain a longer-term objective, requiring repeated feedback operation and a sustainable energy balance across sensing, processing and actuation.

### 7.4. Materials, Manufacturing, and Human-Centered Translation

Translation beyond laboratory demonstrations requires reliability to be considered at the level of the complete device stack. Triboelectric surfaces, encapsulation layers, electrodes, interconnects and stateful electronic components can degrade through different mechanical and environmental processes. Self-healing electronics and mechanically robust triboelectric architectures provide routes toward recovering functionality after deformation or damage [104,123], while transfer printing and hydrogel-based structures expand the available fabrication space for conformable interfaces [124,125]. The relevant endpoint progressively shifts from the recovery of electrical output to the restoration of sensing, processing and ultimately task-level functionality. Translation therefore requires materials, manufacturing, lifetime and human-interface constraints to be addressed at the level of the complete heterogeneous sensory system. Evaluating the sensing–state–readout pathway after repeated deformation and environmental exposure would help establish whether materials recovery also restores functional performance.

Manufacturability and sustainability will increasingly become coupled design requirements. Recyclable and biodegradable triboelectric materials demonstrate that end-of-life considerations can be introduced at the materials stage [126,127]. Sensory-intelligence systems, however, combine triboelectric polymers with electrodes, semiconductors, electrolytes, encapsulants and interconnects, making the lifecycle of the complete heterogeneous stack more complex than that of the triboelectric layer alone. Future materials strategies will therefore need to consider fabrication yield, service lifetime, repairability, disassembly and end-of-life treatment alongside electrical and mechanical performance.

Human-centered translation introduces a further set of constraints for wearable, prosthetic and interactive interfaces. Long-term use requires mechanical comfort, stable calibration and predictable system behavior, while sensory signals derived from touch, movement and gesture can reveal patterns of user activity even without conventional imaging. For wearable interfaces, user-specific calibration and periodic checks of signal drift provide practical starting points for maintaining predictable operation during long-term use. Local processing and event-based sensing may reduce the amount of information that must be transmitted, but human-centered deployment will also require appropriate control over data acquisition, retention and adaptive behavior. Biocompatibility, clinical usefulness and privacy therefore become system-level properties that require evidence appropriate to the intended application rather than being inferred from flexibility, low operating voltage or local computation alone.

## 8. Conclusions

Triboelectric sensory intelligence can be understood as a sequence of coupled physical transformations linking mechanical interaction to electrical representation, internal state evolution, information processing and, ultimately, action. Within this pathway, triboelectric transduction serves as the physical origin of the sensory representation on which subsequent memory, computation and inference operate. System performance is therefore determined by the fidelity with which task-relevant information is encoded, transferred across source–receiver interfaces, transformed by stateful devices and preserved through to the intended sensory outcome.

Charge storage, ferroelectric polarization, ionic and electrochemical dynamics, and resistive switching provide distinct physical state variables spanning different retention timescales and computational functions. These mechanisms enable recent-event memory, temporal weighting, thresholding, nonlinear transformation and persistent state storage close to the sensory interface. Representative achievements include a mechanically induced threshold shift of approximately 3.5 V retained for almost 1 h [5] and eight resistance levels observed over 500 s in a mechanically programmed memory system [59]. Together with distributed sensing arrays, shared-interface architectures and increasingly integrated hardware, they are progressively reducing the physical separation between sensing, state formation and information processing. Sensory intelligence, however, does not require every operation to reside within a single device; its functional value depends on how effectively transduction, state evolution, computation and readout are coordinated across the complete physical pathway. The compared studies provide stronger evidence for local memory and processing than for continuously adaptive, autonomous operation, with task-level perception frequently involving downstream software.

The next stage of the field will be defined by reliable system-level integration across this pathway. Reproducible mechanical encoding, robust source–receiver coupling, scalable and adaptive stateful processing, energy-aware operation and renewed sensing after action will determine whether triboelectric interfaces can progress from isolated device demonstrations to persistent and autonomous sensory systems. Reported values such as 11.9 fJ/spike local dissipation [73] and 1.2 ms encoding resolution [6] demonstrate progress at specific stages, while complete-task energy and sensing-to-action latency remain essential measures of integrated performance. The central opportunity lies in exploiting the intrinsic coupling among mechanical interaction, triboelectric charge and internal device states to create interfaces that physically encode, retain, transform and act on sensory information with clear task-level benefit.

## Figures and Tables

**Table 1:** Scope of related reviews and the contribution of the present Review.

Review	Main Scope	Complementary Focus of This Review
Torricelli and Pace [8]	TENG coupling with synapses and neurons; integration architectures; memory, plasticity and logic.	Relationship between structural integration, physical processing and downstream inference.
Qin et al. [9] and Rameshkumar et al. [10]	Triboelectric–memristive coupling and tactile neuromorphic transducers.	Comparison of different state mechanisms through source–receiver matching and physical implementation.
Ma et al. [12]	TENG–field-effect-transistor interactions for Internet of Things applications.	Charge delivery and electrical loading in relation to state formation and sensory processing.
Li et al. [13], Shrestha et al. [14] and Ding et al. [15]	TENG-based neuromorphic electronics and tribotronic synaptic devices.	Quantitative operating conditions, implementation depth and the scope of reported performance.
Sung et al. [17]	Organic artificial nerves for robotics and bioelectronics.	Comparison with non-triboelectric sensory pathways at the processing and feedback levels.
Zhou and Chai [18]	In-sensor and near-sensor computing architectures and processing location.	Application of processing-location classification alongside structural integration and implementation depth to triboelectric sensory systems.
This Review	Mechanical encoding, source–receiver coupling, state formation, processing, perception and feedback.	Comparison of representative demonstrations by coupling conditions, state dynamics, physical implementation, validation protocols, and energy and latency scope.

**Table 2:** Comparison of mechanical-to-electrical transduction mechanisms.

Mechanism	Transduction Principle	Main Strengths	Main Limitations
Triboelectric [19,20,21]	Contact electrification and electrostatic induction	Contact and motion sensing; mechanically generated excitation	Finite charge; high impedance; contact and environmental sensitivity
Piezoelectric [26]	Stress-induced polarization	Dynamic force and vibration sensing; direct charge generation	Charge leakage limits sustained-load measurements
Piezoresistive [27,28]	Deformation-dependent resistance	Static and dynamic force sensing; simple readout	Electrical bias; drift, hysteresis and creep
Capacitive [29]	Geometry- or permittivity-dependent capacitance	Static and dynamic sensing; low conduction current	Excitation and readout circuitry; parasitic capacitance
Electromagnetic [30]	Motion-induced magnetic flux variation	Rotation and vibration sensing; direct electrical generation	Magnet/coil size and mass; weak output at slow flux variation

**Table 4:** Comparison of representative triboelectric sensory computing demonstrations.

Study	Structural Integration	Processing Location	State Variable and Its Lifetime	Implementation Depth	Physically Measured Versus Computed in Software	Energy Accounting Boundary
Khan et al. [5]	Shared sensing–memory structure: triboelectric PDMS layer gates the MoS_2_ channel.	In-sensor charge storage; external electrical readout.	Surface triboelectric charge; threshold shift up to ~3.5 V retained almost 1 h.	L1	Mechanical writing and retained electrical characteristics measured; no trained classifier.	No external write pulse; powered readout remains.
Sun et al. [58]	Discrete coupling: TENG and resistive memory are separate components.	Near-sensor resistive state update and storage.	Filamentary resistance; nonvolatile switching; lifetime not normalized here.	L1	Mechanical switching measured; electrical SET −0.61 to −0.92 V and RESET +0.365 to +0.875 V distributions.	Read, erase and mechanical writing are separate operations.
Kim et al. [59]	Discrete coupling: TENG, rectifier/resistor and 6 × 6 memory crossbar.	Near-sensor memory-array writing and retrieval.	Interface-type resistance; 8 levels over 500 s; separate >10^4^ s retention test.	L3	Connected mechanical pattern writing and retrieval; no trained task inference shown by the pattern.	TENG write/reset scheme; read bias and peripherals remain.
Kim et al. [61]	Discrete coupling: polymer TENG coupled to a ferroelectric synaptic device.	Near-sensor synaptic state update; off-sensor software recognition.	Ferroelectric polarization; retention depends on pulse history; common lifetime NR.	L2 + H	Source and synaptic characteristics measured; Morse/MNIST network training and recognition simulated.	Electrical weight updates and sensing pulses are distinct; full task energy NR.
Yu et al. [73]	Discrete coupling: TENG coupled to an ion-gel MoS_2_ transistor.	Near-sensor ionic preprocessing; downstream MCU decision and LED output.	Ionic state; sensory-memory regime, stimulation dependent.	L3 + H	Coupled postsynaptic current and MCU/LED logic measured; MCU supplies decision stage.	11.9 fJ/spike is local device dissipation at 1 mV drain bias; back-end and LED costs excluded.
Sultan et al. [62]	Shared triboelectric/electrolyte interface in the ionogel–OECT structure.	Local electrochemical preprocessing; off-sensor random-forest classification.	Ionic/electrochemical state; history dependent, protocol-specific retention.	L2 + H	Mechanical synaptic responses measured; random-forest material classification external.	Drain read bias −0.5 V; whole classification energy NR.
Cho et al. [63]	Discrete coupling: TENGs coupled to a flexible graphene ion-gel transistor.	Near-sensor temporal/synaptic processing; off-sensor software HAR evaluation.	Ionic memory; ~70–450 ms EPSC decay in electrical tests; longer regime >2 s.	L2 + H	Device dynamics measured; hardware-derived weights used with UCI inertial HAR data.	TENG-derived gate/drain stimulation demonstrated; electrical characterization and software task evaluation have distinct supply boundaries. Complete task energy is not reported.
Yun et al. [38]	Discrete TENG–receiver coupling; biristor and charge-trap synaptic circuitry form the receiving hardware.	Near-sensor spike encoding and physical synaptic weighting; final comparison logic not established.	Integration charge and stored synaptic weight; no common lifetime extracted.	L3; task-related output	Physical spikes and weighted output currents measured for two pitches; integrated final comparison logic not established here.	Sensing supply removal does not quantify total bias, weight programming or readout energy.
Lee et al. [56]	Discrete coupling: four TENG–biristor receptor cells.	Near-sensor spike encoding; off-sensor simulated SNN classification.	Transient integrate-and-fire state; no persistent learned weight in sensor array.	L2 + H	Spikes measured; classification uses simulated SNN, with measured and artificial records.	Self-powered receptor does not establish full inference energy.
Chen et al. [6]	Distributed triboelectric receptors coupled to artificial afferent circuits.	Near-sensor afferent/first-spike encoding; downstream spiking layers evaluated in software.	Transient afferent/neuron state; a common decay or retention lifetime is not specified.	L2 + H	64 receptors and first-spike encoding measured (1.2 ms encoding resolution); downstream task evaluation mixes hardware and software. Resolution is not a state lifetime.	Resolution is not decision time; total task energy not established by negligible idle activity.
Wen et al. [99]	Glove-mounted TENG channels connected to acquisition hardware.	Off-sensor acquisition and software inference; no physical learned state in the transducer established.	Sensor waveform; no shared physical learned state established by classification.	Sensing + H	Gesture signals measured; recognition and command generation occur outside the transducer in software.	Self-powered transduction does not cover acquisition, computer, wireless link or effector.
Xiong et al. [100]	Glove-mounted TENG channels connected to acquisition hardware.	Off-sensor acquisition and CNN inference; no physical learned state in the transducer established.	Sensor waveform; no shared physical learned state established by classification.	Sensing + H	Gesture signals measured; acquired signals classified by a downstream software CNN.	Self-powered transduction does not cover acquisition, computer, wireless link or effector.
Chen et al. [28], non-TENG comparator	Non-TENG comparator: piezoresistive sensor, OEST and robotic feedback hardware.	Near-sensor electrochemical response with downstream programmed robotic control.	Electrochemical state; reported relaxation depends on ion composition and connection.	Comparator; excluded from TENG depth counts	Physical tactile feedback and software control; useful system benchmark, excluded from TENG maturity counts.	External gate/drain and robotic control supplies; no basis for self-powered TENG-system claim.

L1: measured state response; L2: physical processing primitive; L3: connected hardware performing multiple operations. H: downstream software or simulation.

**Table 5:** Learning methods and evaluation protocols in representative triboelectric sensory studies.

Study and Task	Learning Method	Data Partitioning	Performance
Phan et al. [46], surface-potential prediction	CNN, LSTM and TCN regression	Seven training materials with an 80:20 record split; three additional materials held out for evaluation.	Surface-potential regression on held-out materials.
Lee et al. [56], touch-gesture recognition	Software SNN using physically encoded spikes	145 measured records augmented to 1700 training records; independent test partition not specified.	92.5% training-set accuracy; performance on an independent test set was not established.
Wen et al. [99], sign-language recognition	CNN using acquired glove signals	80:20 split for word recognition; 60:20:20 for sentence segmentation; additional tests on novel word combinations.	Word recognition and sentence segmentation evaluated separately.
Xiong et al. [100], seven-gesture recognition	CNN using acquired TENG signals	1400 gesture records randomly divided into training and test sets at 80:20.	98.57% classification accuracy.
Guo et al. [111], vibration identification	1D-CNN using signals from a TENG–synaptic-transistor system	Partitioning protocol not specified in the examined main text.	88.9% classification accuracy.
Xu et al. [30], rehabilitation and interface tasks	CNN using hybrid triboelectric–electromagnetic signals	Random 4:1 splits for several tasks; five-fold leave-one-participant-out validation also reported.	Task-specific accuracy could not be confirmed.

**Table 6:** Energy and timing boundaries in selected headline demonstrations.

Study	Energy Metric	Temporal Metric	Measurement Scope
Yu et al. [73]	11.9 fJ/spike at V_ds_ = 1 mV.	No temporal metric reported.	Local afferent dissipation; back-end processing and LED circuitry excluded.
Chen et al. [6]	Complete task energy not quantified.	1.2 ms encoding resolution; 0.32 s to 62.5% accuracy in the selected curvature/stiffness test.	Receptor encoding and task-specific classification; end-to-end inference and actuation energy not quantified.
Kim et al. [59]	Complete write–read–reset cycle energy not quantified.	≈40 ms conditioned write pulse at ≈4.5 V.	Mechanically driven memory programming; pulse duration describes the write stimulus rather than a complete memory-operation cycle.
Chen et al. [28]	Complete feedback-loop energy not quantified.	≈234 ms rise and ≈12 ms recovery with 0.1 Hz gate pulses from 0 to −0.6 V; 80% TFSI, V_ds_ = −0.3 V.	Electrically characterized OEST response; robotic correction latency not established by these device transients.

## Data Availability

No new experimental data were generated in this review. Data sharing is not applicable to this article.
